# Pragmatics and Social Cognition in Learning and Remembering Words

**DOI:** 10.1162/OPMI.a.383

**Published:** 2026-09-05

**Authors:** Katherine Trice, Dionysia Saratsli, Anna Papafragou, Zhenghan Qi

**Affiliations:** Department of Psychology, Northeastern University; Department of Linguistics and Cognitive Science, University of Delaware; Department of Linguistics, University of Pennsylvania; Department of Communication Sciences and Disorders, Northeastern University

**Keywords:** word learning, pragmatics, social cognition, memory, individual differences

## Abstract

Both children and adults can use sophisticated pragmatic inferences to acquire word meanings. An intriguing hypothesis is that words actively acquired via pragmatic inference (as opposed to simpler and more direct word-to-world mapping) might be better retained over time. On this hypothesis, to the extent that socio-cognitive skills support pragmatic computation, such socio-cognitive skills should also support inferentially-driven word learning outcomes. Alternatively, when word-to-world mapping is learned more directly, certainty during encoding might lead to more robust memory outcome. In the current study, we compared adults’ immediate attainment and later retention of novel word meanings acquired through either direct mapping or pragmatic inference. We found that pragmatic inference facilitated the retention of novel words compared to direct mapping (Experiment 1). Moreover, better socio-cognitive skills were associated with better retention specifically for meanings that were inferred but not for meanings acquired through direct mapping (Experiment 1). Furthermore, when a social cognition task was performed before word learning, the advantage of pragmatic inference for meaning retention was diminished. This effect on word meaning retention was not seen when an inhibitory control task with similar attention demand was performed before word learning (Experiment 2). In conclusion, pragmatic processes supported by social cognition play a facilitatory role in adults’ word meaning retention.

## INTRODUCTION

### Pragmatic Inferences and Word Learning

During referential communication, language learners need to successfully map novel words to target meanings by identifying the appropriate entity that the speaker had in mind within a world full of countless potential referents. In this disambiguation process, learners rapidly take advantage of the linguistic and non-linguistic cues available in the input (Fisher et al., 2010; Gleitman, 1990; Naigles, 1990; Yu & Smith, 2007). This intricate process is driven by the fundamental assumption that a speaker’s linguistic choices reflect their communicative intention (Clark, 1990; Grice, 1975; Sperber & Wilson, 1986). Furthermore, these processes are far from restricted to early language learning; from educational to vocational to social settings, acquiring new words occurs across the lifespan (Kavé, 2024).

There is a wealth of evidence that pragmatic cues pointing to the speaker’s intention shape word learning (Bloom, 2000; Diesendruck, 2005; Diesendruck & Markson, 2001; Tomasello, 2000; for reviews, see Bohn & Frank, 2019; Grassmann, 2014; Papafragou & Grigoroglou, 2021). These cues include the interlocutor’s visual perspective (Nadig & Sedivy, 2002), eye gaze (Baldwin, 1993; Nurmsoo & Bloom, 2008), behavior and characteristics (Akhtar et al., 1996; Koenig & Harris, 2005; Sabbagh & Baldwin, 2001; Tomasello & Barton, 1994), as well as properties of the referential scene (Huang et al., 2013; Ryskin et al., 2019; Sedivy et al., 1999) and features of the discourse such as topic continuity across utterances (Horowitz et al., 2018) and continue to be reliably used into adulthood. Such findings show that, depending on the context within which novel meanings are heard, learners can take advantage of pragmatic cues alongside other (e.g., lexical) constraints during referential disambiguation.

It seems plausible that the kind and depth of pragmatic reasoning necessary to reconstruct a speaker’s referential intention might vary across contexts. An ambiguous context with multiple competitors and limited linguistic cues requires a more complex pragmatic computation as learners need to rely more heavily on inference from indirect cues to isolate the target referent in accordance with the speaker’s intentions (Akhtar et al., 1996; Frank & Goodman, 2014; Zosh et al., 2013). Imagine a case where there are two or three toys that have similar characteristics but only one of them has a unique feature that the rest of the toys do not share. Upon hearing a description that contains a novel label (e.g., “I like the toy with the *dax*”), both adults and children are more likely to connect the novel label with the unique feature, assuming that the speaker’s intention is to refer to the feature that is distinctive across these toys (Frank & Goodman, 2014). This assumption is justified if one takes the speaker to aim to be informative: even though it is logically possible for *dax* to refer to a feature shared by more than one toy, this meaning would fail to offer adequate information for the hearer to correctly and unambiguously identify the toy being talked about from among other toys in the scene. To preserve the assumption that the speaker meant to be sufficiently informative, it is thus reasonable to infer that *dax* refers to the distinctive feature.

In contrast, there can be contexts with minimal ambiguity where a more direct correspondence between the referent and the novel label can be created (Axelsson et al., 2012; Golinkoff et al., 1992; Heibeck & Markman, 1987; Markson & Bloom, 1997; Mervis & Bertrand, 1994; Woodward et al., 1994). In such learning contexts, for instance, there can be one novel object, with no other referent competitors in the scene. Therefore, upon hearing an ostensive description (e.g., “This is a *dax*”), learners across the lifespan can successfully encode the meaning-referent association between the novel label and the novel object through a more direct mapping process (Golinkoff et al., 1992; Halberda, 2006; Horst & Samuelson, 2008; Spiegel & Halberda, 2011; Wilkinson et al., 2003). In this process, learners can still make use of socio-pragmatic cues such as a speaker’s pointing or eye-gaze (Graham et al., 2010; Grassmann & Tomasello, 2010; Moore et al., 1999), but the pragmatic computation needed to reach the referent selection is less complex.

To date, very few studies have compared word learning across contexts that invite a direct mapping between a novel label and a referent and those that require a more indirect mapping supported by an inference-intensive pragmatic computation. In one such study, Zosh et al. (2013) compared preschoolers’ novel word learning when the target meanings were either explicitly introduced through instruction or implicitly inferred across the presence of two competitors which required learners to eliminate alternative meanings reserved for other known referents present (a process known as *mutual exclusivity*; see Halberda, 2003, 2006; Markman & Wachtel, 1988; Merriman et al., 1989). During the learning trials, children spent more time looking at the novel target object when this was presented in a direct instruction context rather than in a context that invited an inference. However, when tested after the learning trials, children were more likely to remember the novel meanings that were presented in the inference context rather than the direct instruction context. Thus, despite the availability of the word-meaning link in a direct instruction context, this link was not necessarily better remembered, even immediately after learning.

### Pragmatic Inference and Word Meaning Retention

The focus of the word learning studies reviewed earlier has mostly been directed towards memory tests immediately following learning, not long-term retention, despite the obvious importance of the latter for vocabulary acquisition in the real world. In the Zosh et al. (2013) study, for instance, memory trials were introduced immediately after learning and therefore recall rates did not necessarily reflect long-term memory of the target meanings; in other words, this evidence cannot strongly support the advantage of inferential processes over direct mapping in word meaning *retention*. Studies using directly introduced meanings found a difference between successful, initial referent selection and actual retention of the target mappings over time (Axelsson et al., 2012; Bredemann & Vlach, 2021; cf. Horst & Samuelson, 2008). Success at longer-term retention in both adults and children seems to stem heavily from the engagement of processes that support memory during and/or after encoding processes (Vlach & Sandhofer, 2012). Researchers have capitalized on this by exploring word learning in specific scenarios evoking potential supporting mechanisms such as attention and visual recognition memory (Hartley et al., 2020; Kucker & Samuelson, 2012; Pino Escobar et al., 2023; Vlach & DeBrock, 2017). Could meanings acquired through pragmatic computation similarly serve as one such support and be more resistant to forgetting over longer time lags?

Some studies that did compare direct and indirect mapping contexts have not found a difference in word meaning retention. Jaswal and Markman (2001, 2003) showed that preschool children tested after a two-day delay were equally likely to retain novel labels (common or proper nouns) regardless of whether these labels were learned directly (through ostensive description) or indirectly (through syntactic cues). In other words, preschoolers showed evidence of retaining certain semantic features after a delay, regardless of how the novel label was learned. Moreover, children could update meaning representation for a novel word to a category after a brief encounter of a new referent-label pair. However, it is not clear if the observed retention rates in these findings reflect robust memory representation for specific features rather than the objects themselves. Additional evidence comparing the effects of direct cues vs. pragmatic inference on word learning and retention is currently needed. In contrast, others such as Pino Escobar et al. (2023) offer further evidence for pragmatic computations as a support mechanism. This study found better retention for words mapped via mutual exclusivity, which we conceptualize as a type of pragmatic inference, over those learned statistically via cross-situational word learning. However, none of these studies sought to study the role of pragmatic inferences specifically, let alone in comparison to direct mapping. This limits our understanding of the role that linguistic context may play in supporting word meaning retention. Equally critically, these studies did not tease apart what cognitive mechanisms may underpin any differences in memory advantage seen, leaving our understanding of how these connect to possible memory mechanisms speculative.

### Pragmatics, Socio-Cognitive Skills and Word Meaning Retention

So far, we have assumed that pragmatic computations during word learning involve building a model of the speaker and thus rely on the ability to think and understand other people’s mental states, often referred to as *Theory of Mind* (ToM) (Apperly, 2012; Baron-Cohen et al., 1985; for a review, see Hawthorne-Madell & Goodman, 2019). This position is embedded within models of pragmatics that treat pragmatic inference as a species of intention recognition (Grice, 1975; Sperber & Wilson, 1986). Furthermore, it is supported by studies that link the substantial variation found in ToM abilities, including in adults (Apperly, 2012; Baron-Cohen et al., 2001), to differential performance in pragmatic computations. For instance, Fairchild and Papafragou (2021) found that individual differences in ToM in adults were uniquely associated with pragmatic reasoning: the more sophisticated ToM skills participants had, the better they assessed the pragmatic meanings of scalar implicatures and indirect requests (cf. Matthews et al., 2018). Neuroimaging work, often focusing on adult subjects, also supports the involvement of ToM in pragmatic inference, as brain regions associated with ToM, such as the right Temporo-Parietal Junction (rTPJ) and the medial prefrontal cortex (mPFC) (e.g., Saxe & Kanwisher, 2003) were activated during the processing of various pragmatic phenomena (e.g., Bambini et al., 2011; Bašnáková et al., 2014; Hornick & Shetreet, 2022; cf. Kampa et al., 2025; Spotorno et al., 2012). Because developmental studies have found ToM involvement in early word learning (see, e.g., Brosseau-Liard et al., 2015)—which is in-line with other studies that discuss selective learning in relation to individual children’s differences in socio-cognitive abilities and specifically ToM (DiYanni et al., 2012; Fusaro & Harris, 2008; Sabbagh & Baldwin, 2001; Trice et al., 2025; cf. also Gollek & Doherty, 2016)—it is reasonable to assume that the support of this system in vocabulary acquisition extends into adulthood. However, this has not been empirically tested. Furthermore, even though a wide range of studies has examined the link between word learning and mentalizing, to the best of our knowledge, none of these studies have empirically investigated whether individual differences in ToM are uniquely associated with encoding and—more importantly—retaining novel word meanings (for a similar argument, see Trice et al., 2025).

This is especially critical in adults, as our current understanding of the role of ToM—andeven pragmatics more generally—in word learning is largely limited to developmental work. While adults are capable of making rapid and sophisticated judgements of speaker intent with ease and applying them to linguistic interpretations, the significant individual differences in success across various pragmatic processes indicate that even mature ToM systems are likely to demonstrate salient variation when used (Bašnáková et al., 2014; Fairchild & Papafragou, 2021; Spotorno et al., 2012). Thus, in turn, individual differences in ToM are likely to influence word acquisition across the lifespan.

Evidence for such connections would be particularly important because the role of ToM in word learning and pragmatics has been challenged in some cases, including in adult populations (Andrés-Roqueta & Katsos, 2020; Kissine, 2012; Pijnacker et al., 2009; for discussion, see Papafragou & Grigoroglou, 2025). Take scalar implicatures—interpreting, for instance, the utterance *Some of my friends left* to mean “some but not all”—which are often conceptualized as being interpreted via pragmatic inference driven by informativeness. There have been mixed findings on whether groups known for weaknesses in ToM resolve these inferences similarly to their neurotypical peers (Ferguson et al., 2018; Noveck et al., 2007; Pijnacker et al., 2009; Wampers et al., 2018). However, as several of these studies note, individual differences in ToM skills may be driving these mixed effects. In addition, in populations with strong ToM skills, examining the role that ToM plays by correlating ToM abilities with the immediate pragmatic task may obscure meaningful relationships and thus undercut the role ToM plays. This is especially true when the pragmatic computations are more straightforward and thus there may be limited individual variation in accuracy. By looking beyond in-the-moment pragmatic inferences and considering the retention of word meanings, we may be able to capture effects that would otherwise be missed.

### Current Study

In the current study, we add to our understanding of the role of pragmatics in word learning and retention and the kind of socio-cognitive abilities that support pragmatic inferences. The goal of the study is two-fold. First, we probe whether word meanings acquired through pragmatic-inferential computation are retained better compared to words acquired more directly. Second, we explore how pragmatic-inferential computations that carry this potential word learning advantage are themselves related to individuals’ socio-cognitive abilities such as ToM. Throughout we focus on adult learners since, as alluded to already, the mental lexicon grows throughout the lifespan (Kavé, 2024; Papafragou & Grigoroglou, 2021).

To assess whether pragmatic inference during word learning has an advantage for meaning retention, we compare encoding and retention of novel word meanings across two learning contexts: a context that invites direct mapping between the novel label and the target novel object, and a context in which a more complex pragmatic computation is required in order to infer the label-object link (Experiment 1). One hypothesis is that, if an aspect of pragmatic inferencing drives better semantic encoding, pragmatically inferred meanings should have higher retention rates. Note that this hypothesis is agnostic to the underlying aspect or aspects of pragmatic inferencing—for instance, greater utilization of ToM or active informativeness-based reasoning—critical to this outcome. A competing hypothesis is that if successful retention relies on having readily available meaning-referent connections, then we should expect higher retention rates for meanings that were encoded through a direct mapping process (Koenig & Harris, 2005; Sabbagh & Baldwin, 2001; Tomasello & Barton, 1994).

To investigate the unique contribution of ToM in inferential word learning and retention, we first assess the link between ToM and the ability to retain pragmatically inferred words (Experiment 1). Here, we hypothesize that a correlation between ToM score and retention for pragmatically inferred words will indeed be present. The potential mechanistical link between the two will depend on the pattern of results and will be discussed more thoroughly in Experiment 1: Discussion. However, the findings of Experiment 1 are correlational. To begin to probe causality, we engage socio-cognitive capacity prior to word learning (Experiment 2). We expect that pre-engagement of the socio-cognitive system should impact the memory strength of meanings specifically for those encoded through pragmatic inference.

This leads to our conceptualization of ToM as a both a stable individual trait and modulable, contextually-deployed state. Our assessment of ToM skill captures it on a trait level. However, how this skill is engaged during word learning itself may be contextually-dependent and malleable. As such, correlations between ToM score and word learning outcomes will primarily support ToM as a trait (Experiment 1 & 2). However, to provide evidence specifically for state engagement, we will focus on evidence of the degree of ToM engagement potentially being impacted by prior engagement and sensitive to learning context (Experiment 2)

## EXPERIMENT 1

In Experiment 1, we tested the relationship between pragmatic inference and word learning in adults by manipulating the degree of necessity for inferential engagement during the initial phase of mapping a novel word form onto its meaning. We also took an individual differences approach by associating each individual’s social cognitive ability, measured by a Theory of Mind (ToM) task, with their word learning outcomes. Word learning behavior was measured by successful recall both immediately after learning and 10 minutes later.

### Experiment 1: Methods

#### Participants.

Based on a power analysis (95% power, *α* = 0.05, see Supplementary material for further details), thirty-six adult participants between the ages of 18 and 22 (31 female and 5 male) were enrolled into the study. All participants were monolingual native speakers of English, with no exposure to a second language before the age of 5. They were undergraduate students at the University of Delaware and were compensated with partial course credits for their participation. Informed consent was obtained for all subjects.

#### Stimuli and Procedure.

The experiment was administered asynchronously through the online experimental platform *Gorilla* (Anwyl-Irvine et al., 2020). Participants were sent a hyperlink that transferred them to the host platform to complete the experiment.

The experiment started with a word learning task, where participants were exposed to sixteen novel words divided across two learning contexts (8 unique novel words per context) in a counterbalanced blocked design of one block per context. Their initial recall of the word meanings was assessed immediately after each learning block in a picture-noun matching test. This combined task totaled 8 minutes in length. The word learning task was then followed by two counterbalanced tasks that measured skills of Theory of Mind (ToM, see Theory of Mind (ToM) task) and Executive Function (EF, see Control task). The EF task was included in our design as a control measure to examine the unique effect of social skills on word learning and retention. The experiment ended with a second picture-noun matching test with approximately 10 minutes of delay after the first picture-matching as a measure of meaning retention. This ensures that learned words have already shifted from immediate to delayed memory, and through the lag when individual difference measures are taken, short-term representation for the novel words will have been transferred to memory-consolidation systems (Radvansky et al., 2022). Furthermore, including cognitively engaging tasks in the interim ensures, at minimum, that participants will unlikely to be able to draw from short-term memory, as maintenance strategies such as covert rehearsal would be disrupted.

#### Word Learning Task.

The design of the task adapted and expanded on an earlier paradigm to study pragmatically inferred word meanings from Frank and Goodman (2014)^1^. The task contained two word-learning contexts: direct mapping, in which there was only one novel stimuli with no linguistic ambiguity present in assignment, and pragmatic inference, in which competition between possible referents could only be resolved through pragmatic computation to overcome semantic ambiguity. Participants were first exposed to a practice phase designed to familiarize them with the experimental setup and presuppositions. Meaning acquisition success was assessed in three phases: in-the-moment mapping, immediate recall, and 10-minute retention (Figure 1).

**Figure 1.**
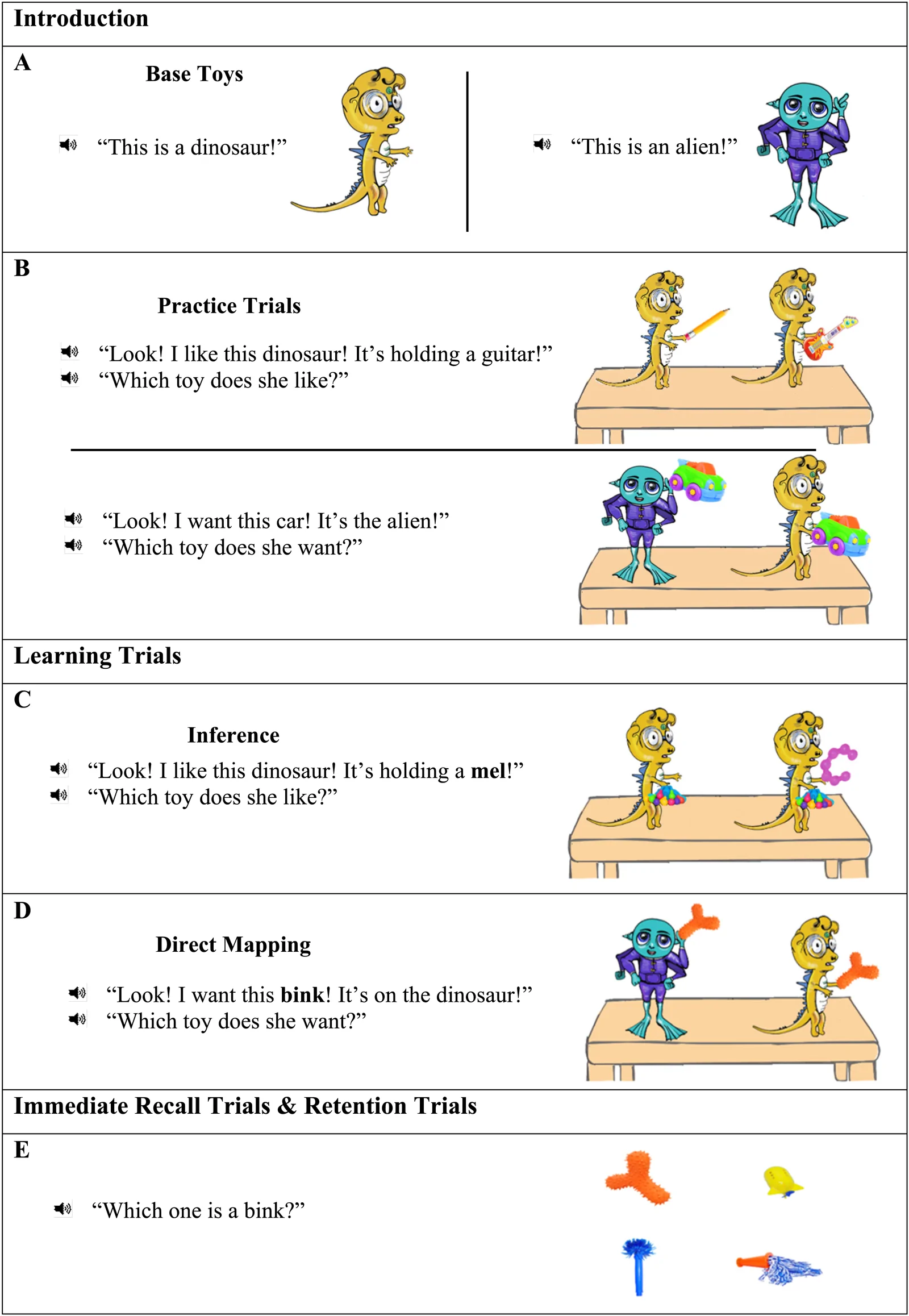
Word learning paradigm with example visual and auditory stimuli (Experiment 1). (C&D) were counterbalanced in a block design, with all words for the condition learned within that block, and each was paired with an immediate recall block (E) testing the just learned words. Retention trials (E) occurred at the end of the experiment after individual difference measures for all learned words.

During the practice phase, participants were introduced to a cartoon tour guide for a toy store tour, as well as the salient base toys (Figure 1 Panel A). Participants were instructed by the narrator to select the toy the tour guide liked or wanted. Critically, participants completed two practice trials that mirror the display setup for each experimental condition but with familiar objects (see Figure 1 Panel B). For the direct mapping condition, this was used to establish the clear framework of semantic resolution to early ambiguity and to reinforce selection of held objects. For the pragmatic inference condition, while the practice trial established that resolution was possible and expected based on the held objects, the stimuli were designed such that an understanding of the practice trial setup cannot be leveraged to resolve later pragmatic inferences.

During learning, participants were introduced to two types of learning contexts arranged in a counterbalanced block design, with eight novel words learned in each block. Both contexts included two toy characters (either a dinosaur and an alien, or two dinosaurs, or two aliens) holding small novel objects and were placed on a table. The locations (left vs. right) and the animal toy type (alien vs. dinosaur) of the target object were counterbalanced across trials. Participants would hear a statement by the tour guide introducing the toys and target object—(1) for an example from the pragmatic inference block and (2) for an example from the direct mapping block. All trials in a given block syntactically followed their example format, with just the novel word and choice of character referent swapped out. After the introduction of the toys, participants would hear a question from the narrator asking them to select the toy mentioned in the sentences.

(1)  Look! I like this dinosaur! It’s holding a *mel*.
(2)  Look! I like this *bink*! It’s on the dinosaur.

Known object trials mirroring the practice trials for the given condition discussed above were interweaved in each block to re-enforce the relevant framework for interpreting the statements.

In the inference context (see Figure 1 Panel C), participants would see two identical aliens or two identical dinosaurs, with one toy holding a novel object (a competitor) and the other toy holding the same competitor object and an additional unique target object. Participants would hear two sentences, as in (1). Here, the final sentence fails to resolve the ambiguity, as on purely a lexical, syntactic, or semantic level the novel word could refer to either target or competitor based on the visual scene. Thus, the listener must rely on pragmatic computation for referential disambiguation. As shown in Figure 1, participants would only be able to map the novel label *mel* to the C-shape novel object after they inferred that *mel* was mentioned to disambiguate the referents and therefore had to be the unique object held by one of the two toys. The underlying assumption driving this pragmatic inference is that the speaker is trying to be informative and believes that they have given enough information to accomplish this (Grice, 1975). Note that this is a linguistic pragmatic inference—non-linguistic gestural cues cannot be relied upon to assist in disambiguation, as is common in prior work, and the participant is never informed as to whether they selected the correct referent, maintaining the pragmatic ambiguity and thus need for inferencing throughout the remaining trials.

In the direct mapping context (see Figure 1 Panel D), participants would see one alien and one dinosaur holding the same novel object and they would hear a phrase such as (2) above. The final sentence allows participants to directly map the novel word onto the sole novel object, resolving any ambiguity and thus removing the necessity to make an effortful and uncertain computation as to the speaker’s intent to accomplish this mapping. This is not to suggest that listeners may not engage ToM systems during processing. Both conditions have the listener identifying an object of desire of the speaker, occurring thus in a setting requiring social engagement. Furthermore, the direct mapping condition especially may raise questions of speaker motivation—why they desire one specific novel object over its identical counterpart. Instead, what is being contrasted between conditions is the use of pragmatics and ToM in the mapping of the novel word itself. These features are *incidental* rather than central within this direct mapping, allowing us to control for possible effects of baseline social engagement and the presence of ambiguity—temporary though it may be—on autonomic processes such as attention.

Each of the eight novel words per learning block was repeated twice in two different learning displays held by different toys. In addition, four known object trials were created for each of the two conditions. Therefore, for each learning block, there were sixteen critical learning trials and four filler trials ordered randomly for each participant. Both novel and known words and objects were unique to their given condition. Novel words and object images were obtained from the Noun Database Project (Horst & Hout, 2016). Each object name was randomly paired with one novel object and the name-object pairs were kept identical within each learning context and across participants. Eight additional novel objects were selected as competitors. Eight familiar object images, freely available online under no copyright (e.g., apple and toy car) were included for the practice and filler trials. Success in choosing the correct referent was used as a measure of in-the-moment mapping for the Inference condition.

At the end of each learning block (see Figure 1 Panel E), we tested the immediate recall accuracy via novel word recognition of all eight novel words in eight trials in a forced choice task. Within each test trial, participants saw pictures of 4 objects (in a 2 x 2 arrangement). The three foils and the target were the novel objects presented in the preceding block of learning trials. Participants were asked to identify the correct referent for the novel word upon hearing a question, such as “Which one is a *bink*?”. Retention was tested in the same task 6–10 minutes later, as prior research has shown that words should already have shifted from short-term memory by this point (Radvansky et al., 2022) and intervening tasks would have further disrupted any short-term memory storage. All sixteen novel words are randomly ordered. The foils were a mix of novel objects across the two learning contexts.

All the pictures were edited for brighter colors and higher saturation to increase their saliency within the task’s visual setting. All selected words and objects can be found at https://osf.io/qy7bg/overview?view_only=830246afb8944a248b0a1155cb2be269.

All the auditory stimuli were recorded in a quiet room by two female native speakers of English. One speaker recorded the sentences as the narrator and the other speaker recorded the sentences as the tour guide, with speaker role being kept constant across participants. Subsequently, we edited the sound files to remove any white background noise and set identical intensity levels across all sentences at 70 dB. All sound edits were performed in *Praat* (Boersma & Weenink, 2018).

#### Individual Difference Tasks.

##### Theory of Mind (ToM) Task.

We adapted the *Reading the Mind in the Eyes* task (Mind-in-the-Eyes, created by Baron-Cohen et al., 2001) for a self-administered web-based task, which occurred between the final immediate recall and retention blocks and was counterbalanced with the EF task (see Control task). In each trial, participants were presented with a picture showing a different set of eyes for each trial and below that, participants were provided with four different words, each describing a feeling or mental state. Participants were asked to choose the word they thought best described what the person pictured in the photo was thinking or feeling. There were 36 trials in total presented in a randomized order across participants. The total number of correct responses was computed as a measure of ToM (*M* = 22.47, *SD* = 4.25, range = 14–30). The proportion of correct responses is also reported in Appendix Table A1 to allow for ease of comparison across experiments.

We selected the *Mind-in-the-Eyes* task for two reasons. First, this task is largely regarded as one of the most widely used tasks for assessing individual differences in understanding others’ mental states, being challenging enough to capture individual differences even in adulthood and with good stability over time within individuals (Fernández-Abascal et al., 2013; Greenberg et al., 2023; NIMH, 2016; Pinkham et al., 2014; Vellante et al. 2013). Second, the performance of this task has been associated with adults’ ability to use linguistic pragmatic cues for comprehension as well as their ability to reason about other’s underlying intent and beliefs (Fairchild & Papafragou, 2021; Rosenthal et al., 2019), which are all highly relevant to our experiment. This is unsurprising; *Mind-in-the-Eyes* is a measure of mental states inference from salient cues, just as in pragmatic inferences and other ToM phenomena, and as such makes use of the same core ToM system (Feng et al., 2021; Saxe & Kanwisher, 2003; Schurz et al., 2014; Spotorno et al., 2012; van Ackeren 2012). Since *Mind-in-the-Eyes* requires a different kind of mental-state inferencing from our paradigm while drawing on the same core system, a correlation between this task and our word- learning task would suggest a more domain-general, rather than task-specific link, between ToM and word learning. We note, though, that there are mixed reports, especially in more recent work, on the degree of reliability and validity evidence for the *Mind-in-the-Eyes* task (see Greenberg et al., 2023 & Olderbak et al., 2015 for examples validating the task; see Higgins et al., 2024 & Yeung et al., 2024 for examples questioning the value of the task), As such, we will report internal reliability metrics, in the form of split-half reliability and alpha values, for this task within Appendix Table A3.

##### Control Task.

As a control task, we used the Flanker task that targets participants’ inhibitory control, i.e., their ability to suppress responses that are inappropriate in a particular context. This allowed us to ensure that any correlations seen with ToM reflected neither purely greater generalized cognitive skill nor executive functioning (EF) skills in particular, as the latter is often implicated alongside ToM in pragmatic processing (Apperly, 2012; Carlson & Moses, 2001; Fairchild & Papafragou, 2021; Hughes & Ensor, 2007). Note, as an important caveat, that inhibitory control is one of many EF skills and thus the concurrent impact of others is not ruled out by our task’s inclusion. In our paradigm, we adapted the built-in Flanker task version available from the *Gorilla* platform task inventory. It occurred between the final immediate recall block and retention blocks and was counterbalanced with the ToM task (see Theory of Mind (ToM) task). In each trial, participants were presented with 5 fish, either looking all towards the same direction (left or right; congruent trials) or in different directions (incongruent trials). Participants needed to press the button that matched the direction towards which the fish in the middle was looking, inhibiting the direction of the surrounding fish. Before each trial started, a fixation point remained in the middle of the screen at three different duration times before the fish appeared: 400 ms, 800 ms and 1200 ms. The 36 trials were evenly distributed across these fixation duration rates (12 trials for each duration) and the presentation order of the trials was randomized for each participant. For this task, we used the difference score between participants’ reaction times for congruent and incongruent trials as a measure of their performance (*M* = 22 ms, *SD* = 45.17 ms, range = −109 to 107 ms). Flanker reaction times less than 150 ms were dropped from the analysis to account for the time it takes to plan an action after visual processing for the former (Krigolson et al., 2015). Trials with reaction time greater than 1200 ms were also removed as outliers. 94.92% of the data remained for final analyses. The descriptive statistics of the congruency effect are listed in Appendix Table A1 to allow for ease of comparison across experiments.

#### Transparency and Openness.

We report all data exclusionary/inclusionary criteria, all manipulations, and all measures in the study, and the study follows Journal Article Reporting Standards (JARS) where applicable (Appelbaum et al., 2018). All data, analysis code, and research materials are available at https://osf.io/qy7bg/overview?view_only=830246afb8944a248b0a1155cb2be269. Data were analyzed using R, version 4.2.3 (R Core Team, 2021), lme4 package, version 1.1-32 (Baayen, 2008; Baayen et al., 2008), splithalf package, version 0.8.2 (Parsons, 2021), r2glmm package, version 0.1.2 (Edwards et al., 2008), and the package ggplot2, version 3.4.1 (Wickham, 2016).

#### Analysis Plan.

General descriptions of the analyses and models used will be available throughout 2.6.1’s sub-sections. Further details can be found in “Analysis Details” of Supplementary Materials.

##### Pragmatic vs. Direct-Mapping Contexts.

For learning, participants’ performance between the two contexts—direct mapping and pragmatic inference—will not be directly compared. This is because it was, by design, much easier to disambiguate the referent in the direct mapping condition (e.g., the *bink* that is on the dinosaur, as opposed to the one on the alien) than the inference trials (e.g., the dinosaur that is holding the unique object *mel*). Instead, trial-by-trial generalized linear mixed effects models (*glmer*) for each context will be built to examine mapping accuracy, with random intercepts for items and participants. Split-half reliability will be calculated for inferred words, and the spearman-brown corrected reliability estimate reported.

Subsequently, each item for each participant will be binarily coded learned as learned—if participants chose the correct referent in *both* learning trials—or not learned—if one or both trials were incorrect and thus at or below chance. These learning scores will be used as a predictor in immediate recall and retention models.

To test the effect of learning context on participants’ immediate recall accuracy of the novel words, we will build trial-by-trial generalized linear mixed effects models. Here, context type (inferred vs direct mapping, contrast coding: −0.50, 0.50) will be the fixed effect and learning score as the covariate. Random intercepts are included for items and participants and random slopes for context types. Odds ratios will be additionally calculated. Descriptive statistics for accuracy for all experiments, as well as split-half reliability, can be found in the Appendix Table A2.

A similar generalized linear mixed effects model was built to predict participants’ performance in retention. Here, the only difference will be that trial-by-trial retention accuracy is the dependent variable rather than trial-by-trial immediate recall accuracy.

Descriptive statistics for accuracy for immediate recall and retention for all experiments, as well as their split-half reliability, will be reported in Appendix Table A2. All parameter estimates for immediate recall and retention will be reported Appendix Table A4, with main findings reported in the text.

##### ToM and Memory.

To determine whether individuals’ ToM skills uniquely explain variance in meaning recall and retention after Executive Function (EF), in the form of inhibitory control, is accounted for, we will build separate multiple linear regression models for each memory stage (immediate recall or retention) and for each learning context (direct mapping or inference). Note that these will not be trial-by-trial models but will instead predict the proportion of correct responses. Here, in addition to ToM scores and EF scores as predictors, we will control for participants’ performance on the immediate recall for each retention context. All parameter estimates can be found in Appendix Table A5, with main findings reported in the text. Scatterplots of ToM-Memory and EF-Memory relationships can be found in Supplementary Materials 2.1.

### Experiment 1: Results

#### Pragmatic vs. Direct-Mapping Contexts in Word Learning and Retention.

During the learning phase, participants performed significantly above chance, as indicated by a significant intercept, for both direct mapping and pragmatic inference (M_DirectMapping_ = 0.99, *SD* = 0.11, *β* = 9.58; M_Inference_ = 0.78, *SD* = 0.41, *β* = 2.37; *p*’s < 0.001), suggesting participants were paying attention and were successful in mapping inferred meanings to novel words. Split-half reliability for inferred words was calculated to be 0.91 (95% CI = 0.86−0.95), indicating a high degree of within-subject consistency on the chosen resolution to the pragmatic inferences.

As a reminder, a binary learning score was subsequent assigned to each item for each participant based on whether they selected the correct object both trials of the item (1) or instead performed at or below chance (0).

For immediate recall, our results showed a significant effect of context on immediate recall performance (*β* = 0.84, *SE* = 0.41, *z* = 2.05, *p* < 0.05, OR = 2.34, CI_OR_ = 1.04–5.25; Figure 2). As expected, success during the learning phase significantly predicts better immediate recall (*β* = 1.17, *SE* = 0.35, *z* = 3.33, *p* < 0.001, OR = 3.23, CI_OR_ = 1.62–6.45).

**Figure 2.**
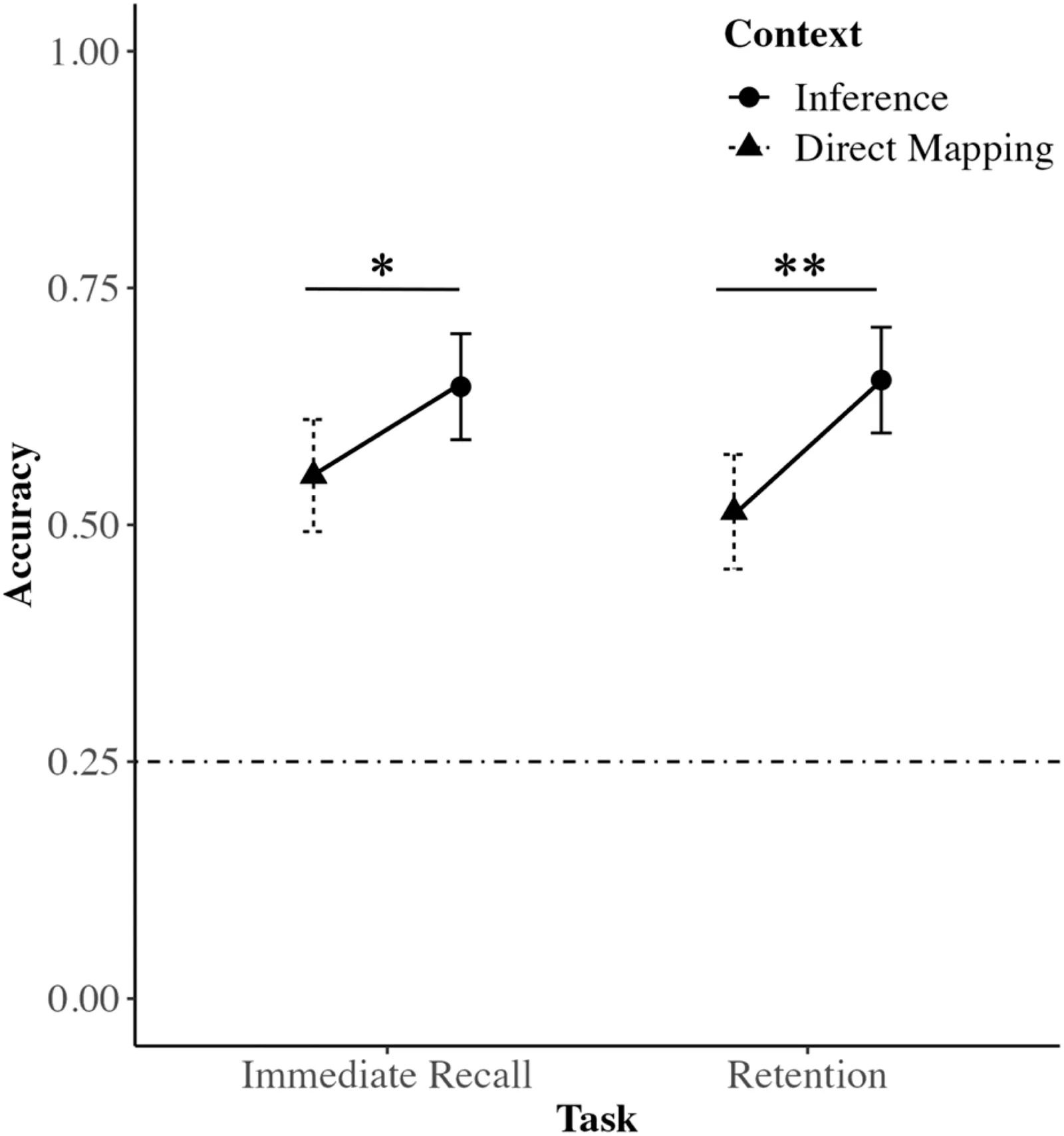
**Proportion of correct responses in the immediate recall and retention for the inferred and directly mapped words (Experiment 1).** Error bars represent the within-subject standard error. The horizontal dashed line represents chance. Significance level: . *p* < .1, *p < .05, **p < .01.

For retention, participants retained inferred words better than words learned in the direct mapping context (*β* = 1.02, *SE* = 0.35, *z* = 2.90, *p* < 0.01, OR = 2.78, CI_OR_ = 1.39–5.54; Figure 2). Learning score also significantly predicted retention (*β* = 1.08, *SE* = 0.33, *z* = 3.30, *p* < 0.001, OR = 2.96, CI_OR_ = 1.55–5.63). Context effects are visualized in Figure 2.

#### Relationship Between Theory of Mind (ToM) and Word Memory.

Next we asked whether individuals’ ToM skills uniquely explain variance in meaning recall and retention after Executive Function (EF) of inhibitory control is accounted for. Note that ToM and EF scores were not significantly correlated with each other, and thus there are not major concerns of multicollinearity (*p* = 0.57).

Results revealed that ToM scores significantly predicted participants’ retention only for words learned through an inference context (*β* = 0.02, *SE* = 0.01, *z* = 2.32, *p* < 0.05, *β* = 0.02, OR = 1.02, CI_OR_ = 1.00–1.03). Moreover, immediate recall was also a successful predictor for participants’ retention accuracy within each learning context (Inference: *β* = 0.66, *SE* = 0.12, *z* = 5.36, *p* < 0.001, OR = 1.96, CI_OR_ = 1.52–2.53; Direct Mapping: *β* = 0.71, *SE* = 0.13, *z* = 5.36, *p* < 0.001, OR = 2.03, CI_OR_ = 1.55–2.66). Similar trends of a nonsignificant relationship were found between ToM and memory for the words learned in the direct mapping context (*β* = 0.02, *SE* = 0.01, *z* = 1.72, *p* = 0.10, OR = 1.02, CI_OR_ = 1.00–1.03). EF scores were not a significant predictor for any model (*p*s* > 0.10).* To better interpret the relative contributions of immediate recall, ToM, and inhibitory control for retention, we examined the semi-partial correlations as indices of their unique contributions to the variance explained by the models. Across both inference and direct mapping contexts, ToM contributed substantially more than EF (Δ*R^2^* Inference: Immediate Recall = 0.61, ToM = 0.26, EF = 0.12; Δ*R^2^* Direct Mapping: Immediate Recall = 0.63, ToM = 0.20, EF = 0.07).

### Experiment 1: Discussion

Experiment 1 investigated the contribution of pragmatic inference to meaning acquisition and retention. We found that adults successfully recalled novel meanings *immediately* after the learning task regardless of whether they acquired them directly or through pragmatic inference. However, the meaning of the novel words that were learned through a complex pragmatic inference was better retained, with this gap in relative retention increasing in the later retention task.

These results show that engaging in a pragmatic computation while creating the target referent-label connection has a beneficial effect in consolidating this connection. To illuminate the potential underlying mechanism, our individual difference analyses suggest socio-cognitive skills play a specific and unique role in the retention of pragmatically inferred meanings. Here, there are three likely accounts. First, an active control/active encoding account, wherein greater cognitive engagement and manipulation during learning would create a stronger memory link (Halberda, 2006; Markant et al., 2016; Ruggeri et al., 2019; Voss et al., 2011). This would align with our findings that pragmatically inferred words are remembered better than those directly mapped. However, this would also predict a negative correlation between ToM skill and retention of pragmatically-inferred meanings. If the amount of general inferential effort needed for the computation by the individual underlies the memory boosting effect (see General Discussion for further consideration), individuals with greater ToM skill would spend less active effort during encoding and would therefore benefit less than those who have less ToM skill and had to work harder. Additionally, the lack of relationship between EF and meaning retention does not entirely rule out active control as a plausible explanation but does make it important to consider other possibilities. Second, if ToM affects memory by easing the computational burden during encoding, then there would be a positive correlation between ToM skill and retention of pragmatically inferred meanings. Literature like Fairchild and Papafragou (2021) have linked better ToM skills to greater success at pragmatic processing. It is possible that less cognitive demand during encoding could lead to better long-term memory (Camos & Portrat, 2015; Jarjat et al, 2018). However, there would be no explanation stemming from this for why pragmatically-inferred words are remembered better than those directly-mapped if reducing the cognitive load plays an important role. Third, the social memory privilege account, that is socially-encoded information is better remembered, would predict a positive relationship between ToM skill and retention of pragmatically-inferred meanings as well as a boost in memory for pragmatically-inferred over directly-mapped words, aligning with our current pattern of results. This would be in-line with non-linguistic literature that suggests that social-cognitive engagement during learning may support longer-term retention (Harvey et al, 2007; Jimenez & Meyer, 2024; Lin et al., 2019). This is additionally supported by the effect of ToM relating to meaning retention, but not immediate recall, and being independent from and contributed beyond the effect of EF within the domain of inhibitory control. These findings imply that the advantage of meaning retention in the inference context might be due to an engagement of social cognition. Furthermore, as our EF task specifically focused on inhibiting differing competitors, this lack of correlation with retention and similar levels of proportion of variance explained across context implies that the pragmatic inference advantage doesn’t stem fully from asymmetries in the visual setup—namely, the pragmatic inference condition has two different novel objects, while the direct mapping condition has two identical novel objects.

If, indeed, ToM engagement, and not just static ability, supports better memory, it would be expected that this is a potentially causal relationship that is malleable based on the degree of concurrent ToM capacity and use. However, the correlational nature of the data in Experiment 1 is insufficient to address the causal role of social cognition in meaning retention. We further begin to tease out potential unique causal effect of ToM skills on the retention of inferentially computed word meaning in our next experiment.

## EXPERIMENT 2

In Experiment 2, we aimed to preliminarily assess the causal role of social cognition in the retention of pragmatically inferred meanings by engaging these skills prior to learning. Previous research found that extralinguistic cognitive functions can be modulated and further modulate language production and comprehension. For example, social affective experiences in a nonverbal video game had an impact on how likely children were to align their lexical choices with conversational partners (Hopkins & Branigan, 2020). In another study, engaging cognitive control with a Stroop task appeared to modulate adults’ and children’s behavior during real-time syntactic disambiguation (Hsu & Novick, 2016; Ovans et al., 2018, 2022). Furthermore, stimulation of the right Temporo-Parietal Junction, which is closely linked with mentalizing, can inhibit processing of context-based linguistic inferences (Feng et al., 2021). Here, we ask whether engaging mentalization prior to word learning has an impact on retention for the newly learned words, especially those learned through pragmatic inference. Our pre-learning ToM task, Mind-in-the-Eyes, is designed to be difficult even for adults with fully mature ToM systems in order to ensure that individual differences in accuracy are readily captured, increasing the lileyhood of heavy engagement of the social-cognitive system prior to word learning. Furthermore, based on work in other domains such as cognitive control (Smalle et al., 2022), the level of real-time engagement of the social-cognitive system during word learning likely depends on the available capacity of this system. Thus, if ToM is indeed an active and necessary component in modulating pragmatic inference retention, we would expect that heavy pre-engagement of the system would alter the within-subject difference between direct mapping and pragmatic inference retention. Note that we are focusing on if there is a difference relative to baseline rather than directionality, as the broader literature has found both boosting and taxing effects of engaging a relevant cognitive system (Hsu & Novick, 2016; Smalle et al., 2022). Our interpretation of the effects of this directionality, if present, will instead be a point of discussion.

Experiment 2 compared word learning and retention in a group of participants who performed the full version of Mind-in-the-Eyes task prior to word learning (ToM-full-First), a new group of participants who completed the same design as Experiment 1 (Learning-First), an active control group who performed a Flanker task prior to word learning (EF-First), and a shortened version of the Mind-in-the-Eyes task before word learning (ToM-partial-First). We designed the last experimental group, as a comparison for the ToM-full-First group, to investigate a graded effect of mentalization engagement. This group performed a partial version of the Mind-in-the-Eyes task prior to word learning. Note that these were blocked designs, rather than our modulating tasks being interweaved with word learning.

### Experiment 2: Methods

#### Participants.

One-hundred-and-forty-four adult participants (18-22 years old, 109 females, 31 males, and 4 who identified as another gender), all native speakers of English, with no exposure to a second language before the age of 5, were recruited and randomly assigned into four experimental groups (Learning-First: *N* = 36, ToM-full-First: *N* = 36; EF-First: *N* = 36, ToM-partial-First: 36). Sample sizes were chosen to match Experiment 1. Participants were a mix of undergraduate students at the University of Delaware, who received partial course credit for their participation, and Prolific members who were similarly enrolled in an undergraduate institution at the time of study completion, who received $5 in compensation (Delaware: *N* = 79, Prolific: *N* = 65). In both cases, participants completed the study online without experimenter supervision. None of our individual difference measures (Mind-in-the-Eyes, Flanker, or Wisconsin-Card-Sorting) differed significantly between those collected via the University of Delaware and those collected via Prolific (*p’s* > 0.05). Informed consent was obtained for all subjects.

#### Stimuli and Procedure.

All stimuli and tasks were kept identical to those of Experiment 1 but task order varied. The Learning-First group, as in Experiment 1, began with the word learning task. The ToM-full-first group completed a full version of the Theory of Mind (ToM) task (Mind-in-the-Eyes) before they proceeded to the word learning paradigm. The EF-first group started with the Control task—the Executive Function (EF) task (Flanker task). The ToM-partial-First group completed a shortened version of the ToM task before they proceeded to the word learning paradigm.

To create this shortened version of the ToM task (Mind-in-the-Eyes task), we reduced the number of trials for the task from 36 to 14. This number was chosen to align with the revised Mind-in-the-Eyes task child version (van der Meulen et al., 2017). The new 14 adult trials were selected randomly from the original Mind-in-the-Eyes task, each addressing a unique emotion. All ToM-partial-First participants were thus presented with the same 14 trials in a randomized order.

On cases where one of the individual difference tasks was moved from the block between learning and retention, an additional filler task of similar length—the Wisconsin-Card-Sorting task—was inserted. This task was viewed as incidental to our main analyses.

Following the practice, word learning, and immediate recall phases, each group completed two cognitive tasks before the delayed recall. A schematic representation of the experimental procedure can be seen in Figure 3. The Learning-First group performed the Mind-in-the-Eyes task and the Flanker task, as in Experiment 1. The ToM-Full-First, EF-First, and ToM-partial-First groups performed the same battery of tasks (Mind-in-the-Eyes task, Flanker task, and Wisconsin-Card-Sorting task), only in different orders. The interval between the end of the word learning phase and the retention phase was kept between 6 and 10 minutes.

**Figure 3.**
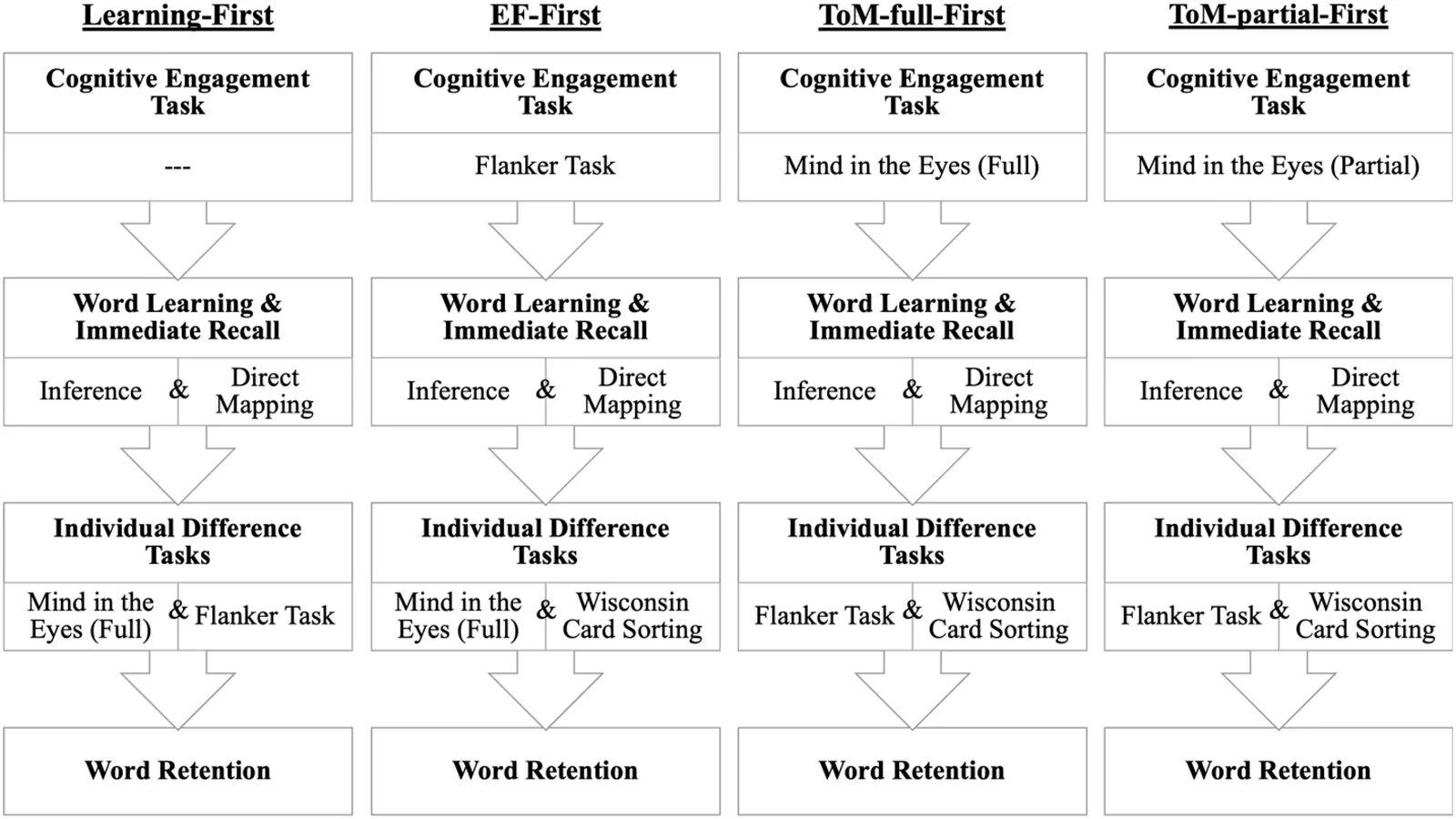
Overall procedure for each experimental group in Experiment 2. *N* = 36 per group. *Note:* For procedural steps with two contexts or tasks listed, the order of these was counterbalanced.

The descriptive statistics of the Mind-in-the-Eyes, Wisconsin-Card-Sorting, and Flanker task performance for each group is provided in Appendix Table A1. For the Mind-in-the-Eyes task, the Learning-First group had significantly greater accuracy than the EF-First group (*p* < 0.05). There was no significant difference between the ToM-full-First group and the EF-First or Learning-First (*p’s* > 0.05). Because the version of the ToM task in ToM-partial-First is different from the other groups, we did not attempt to match performance between this group and the other groups in this task. For the Flanker task and the Wisconsin-Card-Sorting task, none of the four groups for Experiment 2 differed significantly in their scores (*p’s* > 0.05). For Mind-in-the-Eyes, alpha and split-half reliability are reported in for each group in Appendix Table A3.

#### Analysis Plan.

General descriptions of the analyses and models used will be available throughout 3.1.3. Further details can be found in “Analysis Details” of Supplementary Materials.

For each experimental stage—learning, immediate recall, and, and retention—two sets of trial-by-trial generalized linear mixed effects models will be built. The first set will examine between-group differences—ToM-full-First, Learning-First, EF-First, and ToM-partial-First—and the second will examine within-group differences.

For learning, after a sanity check will determine if immediate mappings occurred at an above chance rate (same as Experiment 1). Next, to better understand if ToM or EF engagement prior to the task modulated initial mapping for pragmatically inferred words, we will construct trial-by-trial generalized linear mixed effects model to compare correct mapping rates between our primary group of interest—ToM-full-First (baseline for treatment coding)—and each of our other three groups with random intercepts of items and participants. Where group differences are seen between ToM-full-First and either EF-First or ToM-partial-First, a subsequent model will be created to determine whether including a pre-learning task affects meaning mapping by comparing to Learning-First group. To ensure that any confounding effects of ToM difference between groups were accounted for, secondary, post-hoc models will be created with the inclusion of Mind-in-the-Eyes score as a covariate. In cases where the inclusion of Mind-in-the-Eyes score as a covariate significantly alters the main effects, the findings will additionally be reported. All parameter estimates for between-group models will be reported Appendix Table A6, with main findings reported in the text.

For immediate recall and retention, between-group trial-by-trial models mirror those of learning for between groups, with the addition of context as a main effect and interaction term with group and a random slope of context type. Here, we focus on the interaction between group and context to determine if our pre-tasks differentially altered the relative *degree* of retention success for pragmatic inference vs direct mapping, as there can be incidental baseline differences between groups in terms of their task engagement, language and cognitive skills impacting main effects. However, any main effects will still be reported and discussed. All parameter estimates for these models will be reported Appendix Table A7 for immediate recall and A8 for retention, with main findings reported in the text.

Within-group trial-by-trial models will also be created. These models mirror that of Experiment 1, with fixed effects of context and initial learning, random intercepts of items and participants, and random slopes of context types. As in Experiment 1, initial learning is a binary variable based on whether a given item was selected correctly in both learning trials or was not. Note that these models, during analysis, will be conceptualized as post-hoc, as their goal is to better characterize what patterns of inference vs direct mapping accuracy underpin any significant differences between groups seen. Mean, standard deviation, and split-half reliability values for immediate recall and retention for each group and context can be found in Table A2 in the Appendix. Scatterplots of ToM-Memory and EF-Memory relationships can be found in Supplementary Materials 2.1.

### Experiment 2: Results

#### Learning Contexts and Word Memory.

First, we examined effects within-groups. All groups performed highly accurately during the learning trials for the direct mapped words (> 0.95, *p’s* < 0.001), regardless of which task (Full Mind-in-the-Eyes, Flanker, or Partial Mind-in-the-Eyes tasks) they completed first. Similarly, all groups performed above chance for the inferred words (ToM-full-First: *M* = 0.69, *SD* = 0.43, *β* = 1.23, *p* < 0.01; Learning-First: *M* = 0.72, *SD* = 0.40, *β* = 1.33, *p* < 0.01; EF-First: *M* = 0.80 *SD* = 0.40, *β* = 1.98, *p* < 0.001; ToM-partial-First: *M* = 0.69, *SD* = 0.46, *β* = 0.57, *p* < 0.05). The intercept for each model compared to the chance level is depicted in Figure 4.

**Figure 4.**
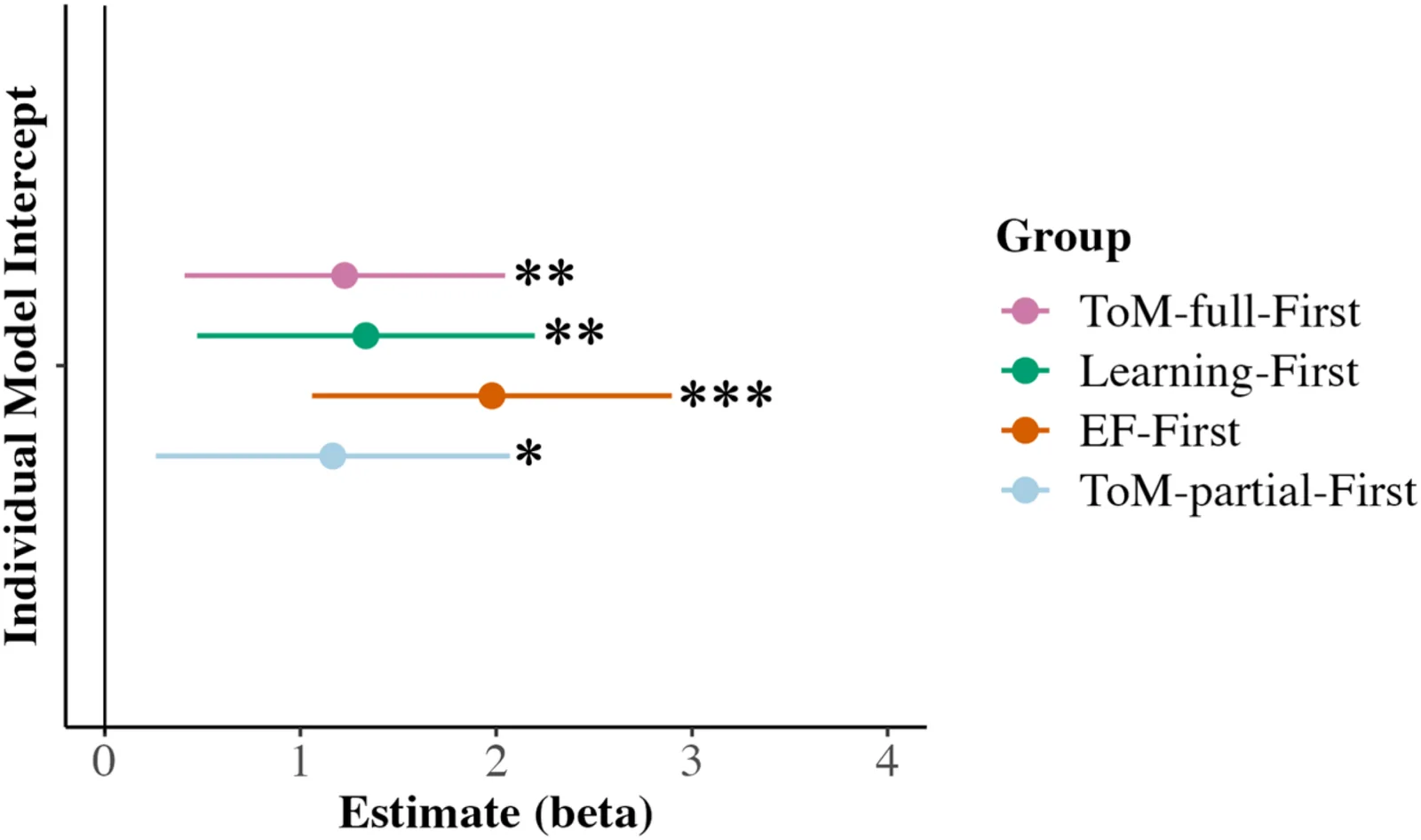
**Model estimates of learning accuracy for the inferred words across Experiment 2.** Error bars represent 95% CI. The asterisks represent within-group significance compared to chance-level. Vertical line represents chance. Significance level: . *p* < .1, *p < .05, **p < .01, ***p < .001.

Then, we examined between-group effects specifically for inferred words. The EF-First group performed marginally worse than the ToM-full-First group; a follow-up analysis with Learning-First as the baseline showed no difference (ToM-full-EF: *β* = 0.83, *SE* = 0.46, *z* = 1.79, *p* < 0.1, OR = 2.29, CI_OR_ = 0.92−5.67; Learning-EF: *p* > 0.10). In contrast, the ToM-full-First and Learning-First and ToM-full-First and ToM-partial-First groups were not different in learning (*p* > 0.1). Our secondary models including ToM as a co-variate did not significantly alter the results. Full model results are reported in Appendix Table A6.

We next examined immediate recall differences between groups. We focus on the interactions between groups and context. None were seen (*p*s > 0.1). We found that there was a significant main effect of group between ToM-full-First and Learning-First, primarily driven by higher accuracy for Learning-First in the inference condition (*β* = 0.53, *SE* = 0.24, *z* = 2.21, *p* < 0.05, OR = 1.70, CI_OR_ = 1.06−2.73). Note that, given that this is an overall accuracy difference across groups, conclusions of whether this difference stems from the manipulation, group differences in memory capacity, or group differences in possible salient factors such as ToM ability is uncertain. No other group differences were seen (*p*s > 0.1). As such, a follow-up analysis with a Learning-First baseline was not motivated. Notably, for our secondary model including the Mind-in-the-Eyes score as a co-variate, the main effect of group is lost while a significant effect of ToM Accuracy emerges (ToM-full-Learning: *p* > 0.1; ToM Accuracy: *β* = 1.92, *SE* = 0.80, *z* = 2.41, *p* < 0.05, OR = 6.85, CI_OR_ = 1.43−32.7). Full model results are reported in Appendix Table A7.

Post hoc analyses with our within-group models further illuminated our results. Here, an advantage was seen for pragmatically inferred words in the Learning-First Group, but not the ToM-full-First Group (Learning-First: *β* = 0.90, *SE* = 0.36, *z* = 2.50, *p* = 0.01, OR = 2.46, CI_OR_ = 1.21−5.00; ToM-full-First: *β* = 0.01, *SE* = 0.33, *z* = 0.04, *p* = 0.97). Thus, while no interaction is present, the group difference seen is likely primarily driven by the effect—or lack of it—of contrast. No context effect was seen for the EF-First Group (*β* = 0.59, *SE* = 0.36, *z* = 1.64, *p* = 0.10), though the context effect was numerically more similar to the Learning-First Group (Inference > Direct Mapping). For the ToM-partial-First group, the context effect was very similar to that of the Learning-First group (ToM-partial-First: *β* = 0.88, *SE* = 0.38, *z* = 2.33, *p* = 0.02, OR = 2.42, CI_OR_ = 1.15−5.08). Thus, despite the lack of difference in our between-group model, the ToM-partial-First group preserves the advantage of pragmatic inference not seen in the ToM-full-First group. Accuracy can be seen in Figure 5A and within-group context contrasts are plotted in Figure 5B.

**Figure 5.**
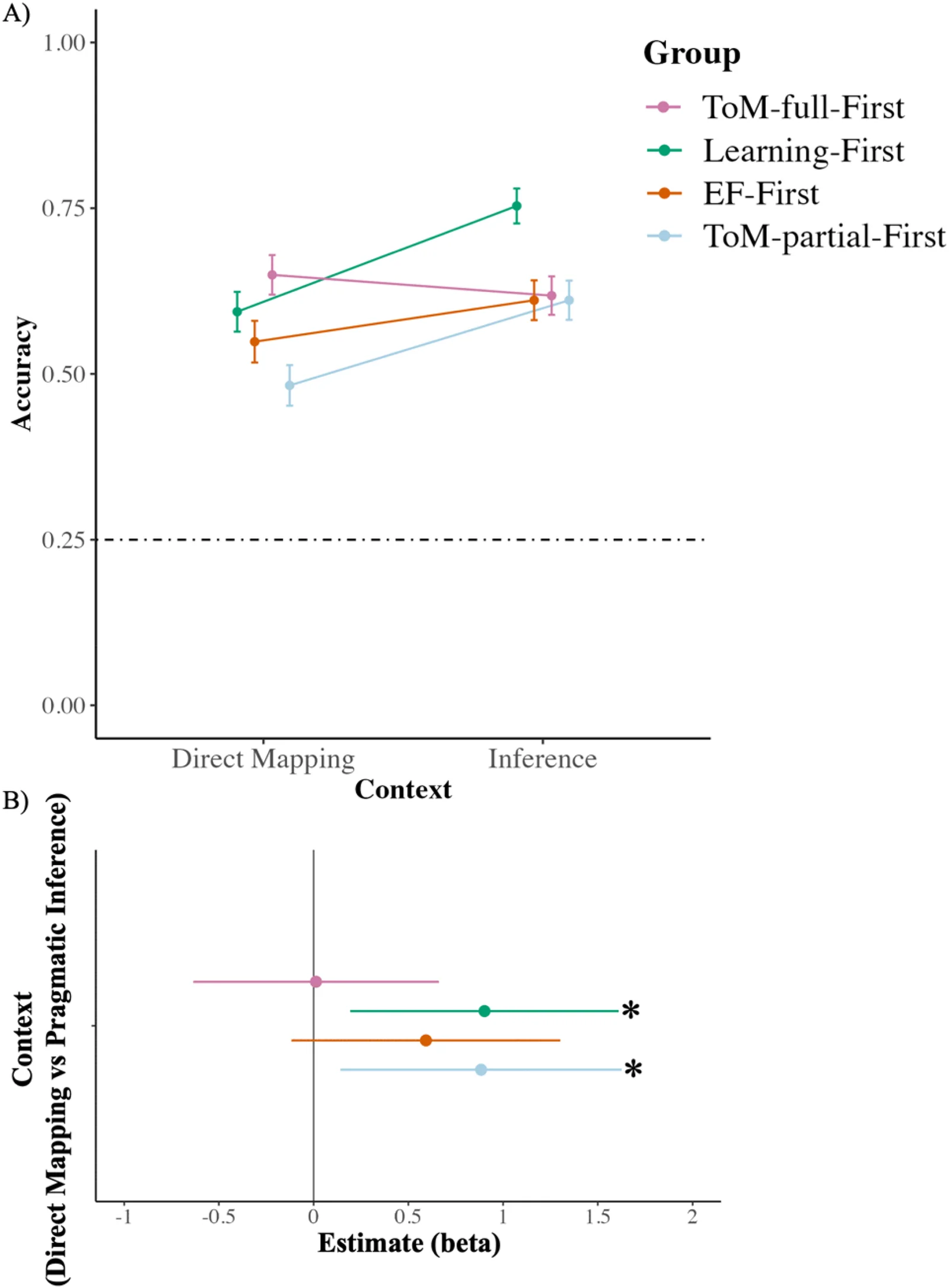
**Immediate recall for directly mapped and inferred words for groups across Experiment 2. (A) Immediate Recall Accuracy.** Error bars represent the within-subject standard error. The horizontal dashed line represents 0.25 chance level. **(B) Model beta estimates of within-group context effects.** Error bars represent 95% CI. The vertical line represents a lack of context effect, with negative numbers indicating the pattern Direct Mapping > Inference, and positive numbers indicating the pattern Inference > Direct Mapping. The asterisks on the right represent within-group significance for the effect of context type. Significance level: . *p* < .1, *p < .05, **p < .01, ***p < .001.

Finally, we examined the Retention task. We focus on the interactions between groups and context. The ToM-full-First group, compared to the Learning-First group (*β* = 0.74, *SE* = 0.35, *z* = 2.13, *p* < 0.05, OR = 2.10, CI_OR_ = 1.06-4.17), the EF-First group (*β* = 0.77, *SE* = 0.34, *z* = 2.23, *p* < 0.05, OR = 2.16, CI_OR_ = 1.10−4.23), and the ToM-partial-First group (*β* = 0.72, *SE* = 0.35, *z* = 2.05, *p* < 0.05, OR = 2.05, CI_OR_ = 1.03−4.05), showed a significantly smaller learning context effect. The interactions with EF-First and ToM-partial-First motivated a followup with Learning-First as the baseline. No interactions were seen. While including Mind-in-the-Eyes scores into the models did not change any main findings, individuals’ ToM score served as a significant predictor for retention scores in each model (*β* = 2.42, *SE* = 0.91, *z* = 2.65, *p* < 0.01, OR = 11.3, CI_OR_ = 1.89−67.40). Full model results are reported in Appendix Table A8.

Post-hoc analyses assessing the context effect within each group found similar advantage of the pragmatic inference context in the EF-First (*β* = 0.75, *SE* = 0.28, *z* = 2.64, *p* = 0.01, OR = 2.12, CI_OR_ = 1.22−3.71), the Learning-First group (*β* = 0.61, *SE* = 0.32, *z* = 1.95, *p* = 0.05, OR = 1.85, CI_OR_ = 0.99−3.43), and the ToM-partial-First group (*β* = 0.57, *SE* = 0.28, *z* = 2.07, *p* = 0.04, OR = 1.77, CI_OR_ = 1.03−3.05), but none in the ToM-full-First group (*β* = 0.12, *SE* = 0.40, *z* = 0.31, *p* = 0.76). Accuracy can be seen in Figure 6A and within-group context contrasts are plotted in Figure 6B.

**Figure 6.**
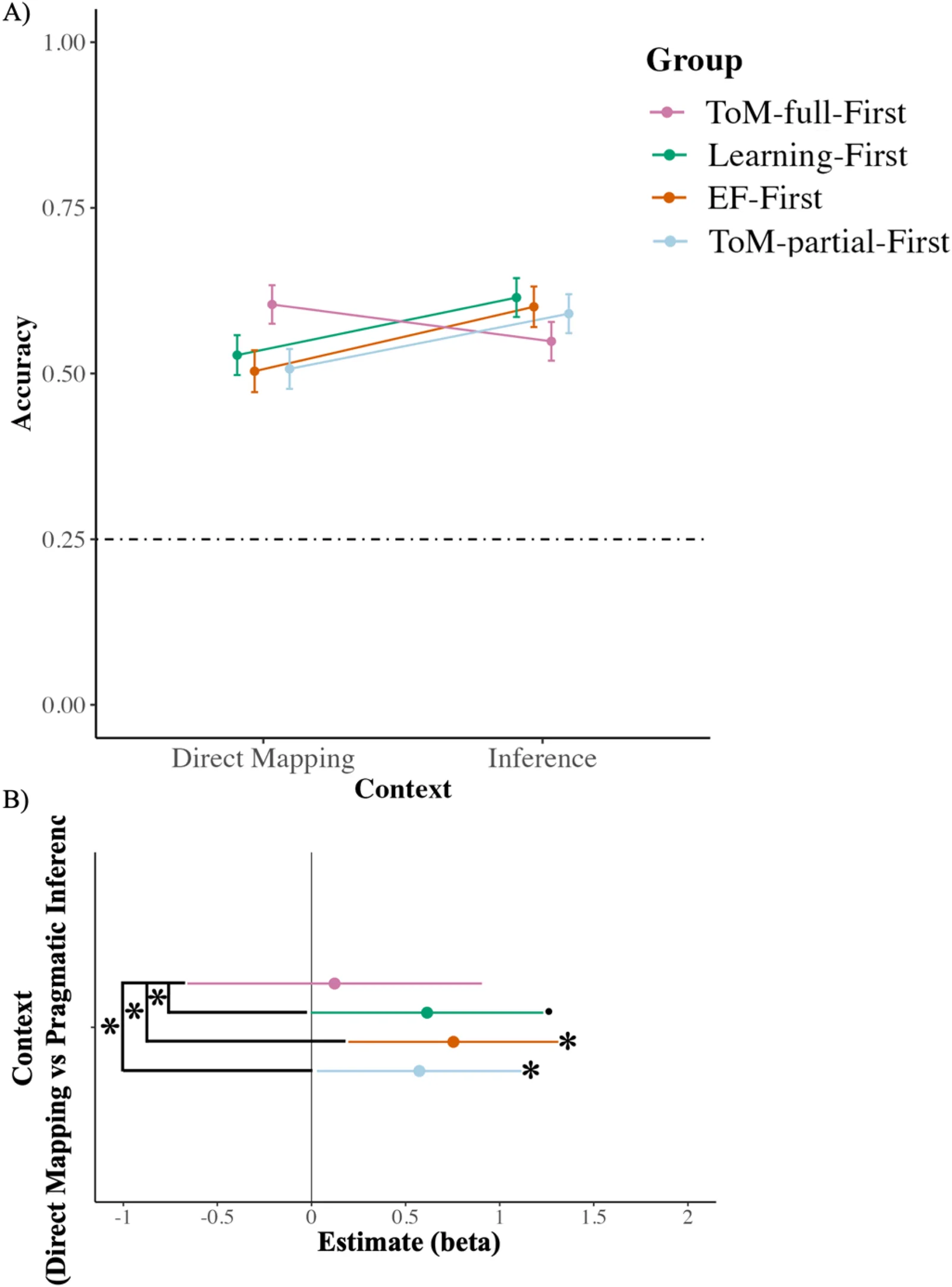
**Retention for directly mapped and inferred words for groups across Experiment 2. (A) Accuracy.** Error bars represent the within-subject standard error. The horizontal dashed line represents 0.25 chance level. **(B) Beta estimates of within-group context effects.** Error bars represent 95% CI. The vertical line represents a lack of context effect, negative numbers indicate the pattern Direct Mapping > Inference, and positive numbers the pattern Inference > Direct Mapping. Asterisks on left represent between-group interactions. Asterisks on right represent within-group significance for context type. Significance level: . *p* < .1, *p < .05, **p < .01, ***p < .001.

### Experiment 2: Discussion

In this second experiment, we aimed to test a potential causal relationship between social cognition and meaning retention by manipulating the availability of social cognition in learners. Here, we manipulated whether, prior to the word learning task, participants completed a Theory of Mind (ToM) task, that taps on their pragmatic thinking, or an Executive Function (EF) task as a control. We replicated the main findings in Experiment 1 with our new Learning-First group and the EF-First group, who showed a similar degree of facilitation from pragmatic inference for meaning retention. These findings suggest that engaging inhibitory control prior to learning does not interfere with the memory advantage by pragmatic inference. However, participants who completed the full ToM task prior to learning showed no benefit of pragmatic inference on either recall or retention. These results indicate that while the taxing effect from the ToM task may not interfere with resolution of pragmatic ambiguities, it may interfere with effective word meaning encoding. Alternatively, the lack of similar boost for pragmatic inference over direct-mapped words may stem from heightened learning for both conditions. The degree of such boosting could be limited for pragmatic inference due to a ceiling effect. We cannot rule out this possibility, given our between-subject design. Memory capacity and task engagement might differ between groups. As such, future within-subject work is critical.

Additionally, we found that a shortened ToM task prior to learning resulted in a similar memory advantage for pragmatically inferred words as the baseline group with no preceding task, suggesting the memory boosting effect of pragmatic inferencing does not depend on a fully available ToM system. This finding has implications for naturalistic generalizations of our work; real-life conversations often require the use of socio-cognitive skills throughout, including emotional identification based on facial expression as in our ToM tasks. Word learning in real-life can still benefit from the engagement of social cognition, even when other socially demanding tasks may compete for the same cognitive resource.

The role of ToM in meaning retention is further bolstered by ToM accuracy emerging as a significant predictor for both immediate recall and retention. This provides further evidence for the importance of ToM trait as a potential supporting factor in memory outcomes, while the lack of condition difference in our ToM-full-First condition provides further weighting for the hypothesis that concurrent state and resources of ToM during mapping have downstream effects for memory. This is supported not only by the fact that modulation being possible marks ToM as malleable but by the effect itself—whether taxing or boosting—being different across contexts.

Overall, Experiment 2 provides evidence for a potentially causal link between learners’ ToM function and meaning acquisition outcomes. Such effects on meaning retention are commensurate with the available social cognition resources during learning and are not similarly impacted by other cognitive tasks such as inhibitory control. This indicates that depth of word encoding may vary with *social* cognitive function beyond in-the-moment resolution.

## GENERAL DISCUSSION

The current study consistently demonstrated a strong association between Theory of Mind (ToM) use for referential disambiguation and superior word meaning retention. The relationship may be specific to words that require pragmatic inferences to acquire because similar facilitation was not observed for the retention of directly-mapped word meanings (Experiment 1). Furthermore, ToM engagement, not just ability, may impact retention, as perturbation of learners’ social cognition, but not domain-general cognitive control, prior to learning had a direct impact on subsequent meaning retention for pragmatically-inferred vs directly-mapped words (Experiment 2). These results provide novel evidence suggesting a lasting influence of pragmatics and social cognition on word learning beyond in-the-moment meaning mappings and have important potential implications for the mechanisms of encoding and consolidation of word meanings in memory.

### Impact of Word learning Context on Memory of Word Meanings

Even though both a direct mapping and an inference context can lead to successful in-the-moment memory encoding (as shown by testing for those meanings immediately after the learning phase), our core finding in this study is a higher retention rate for meanings that were encoded in a context in which learners needed to make a more complex pragmatic computation to infer the target label-referent association. This is in tune with earlier findings suggesting that a direct mapping between the label and the target referent does not necessarily lead to better later retention of the encoded meanings (Axelsson et al., 2012); interestingly, this is true regardless of whether the learners are children or adults. Similarly, in young preschoolers, inferentially acquired words had a better recall rate than those directly mapped onto their meanings (Zosh et al., 2013).

Our findings also align with work that that active control or active encoding during learning creates a stronger memory link for this information (Halberda, 2006; Markant et al., 2016; Ruggeri et al., 2019; Voss et al., 2011). Pragmatically inferring novel meanings requires learners to combine both contextual clues (e.g., there is only one novel object across toys) and the speaker’s communicative intention (that the speaker would make linguistic choices that would precisely guide their listener to their intended meaning in accordance with the principle of informativeness; Grice, 1975). This, in turn, requires a) engaging with all the possible referents available in order to eliminate the irrelevant ones which in turn instigates a more in-depth processing of these elements, especially compared to a one-to-one mapping process, b) constructing and evaluating an internal model of the speaker based on the cues given to compute intentions and c) correctly apply the computed meaning to the available referents to select the correct referent (Craik & Tulving, 1975; Kuhl et al., 2006; Moll & Tomasello, 2004; Tomasello & Barton, 1994; Tomasello et al., 2007; Zosh et al., 2013). Thus, effects of this active encoding would be expected to emerge form pragmatic inference resolution, leading to a more robust representation of the word-object pair in memory. This contrasts with the more passive role that learners have for the direct mapping context: there is minimal pragmatic complexity to drawing a correspondence between the label and the referent since there is only one novel object across both toys.

However, it is important to note that possibility is only partially supported by the current findings. As noted in Experiment 1, active encoding would have the strongest support with higher retention rates for pragmatically inferred words—which is indeed present—*and* a negative correlation between pragmatic inference retention and ToM skills such that individuals for whom the computations are more complex and effortful and thus require greater degree of engagement have better memory. For the latter, we see the opposite pattern of those with better ToM skills showing stronger memory for pragmatic inferences. This is reinforced by the positive correlations between ToM score and memory seen in Experiment 2. However, this does not fully eliminate the possibility of active encoding as a contributing factor. Active encoding isn’t necessarily synonymous with more effortful encoding relative to other individuals. Instead, a theme across studies is that more engaging cognitive processes leading to greater depth of encoding. Thus, this may contribute to the group-level pragmatic inference advantage seen even if greater effort needed may not modulate individual-level results.

Our study thus extends the traditional definition of active learning and encoding to include not just situations where participants have active control over how the stimuli are presented but also situations in which participants actively resolve ambiguities in the stimuli. However, because of the confounding factor of ToM in pragmatic inferences as well as the lack of clear individual modulation, further work is needed to determine if memory advantages for ambiguity resolution have active encoding as a contributing factor. Such work would need to extend beyond pragmatic—or even linguistic—ambiguity resolution and address the degree to which this phenomenon correlates with individual active encoding advantages in other scenarios to determine if similar underlying mechanisms are at play.

### Socio-Cognitive Skills, Pragmatics and Word Meaning Retention

The second major finding in this study linked learners’ social cognitive function with word learning and retention outcomes. We showed that participants’ differential performance in the ToM task, but not a Flanker task, significantly predicted their later retention for words that were pragmatically inferred. In other words, the better participants scored in the ToM task, the better they retained the novel meanings that they needed to pragmatically compute. This pattern is in line with previous research findings that point towards the effect that differential pragmatic skills can have on word learning (Brosseau-Liard et al., 2015; DiYanni et al., 2012; Fusaro & Harris, 2008; Gollek & Doherty, 2016; Sabbagh & Baldwin, 2001). More importantly though, these findings provide novel evidence as to the unique role that pragmatic reasoning can play in relation to successful meaning retention.

In an attempt to further test a potential causal role of social cognition in the retention of inferred meanings, we sought to modulate participants’ ToM function prior to word learning. Participants that started with the ToM task and engaged their pragmatic reasoning skills before the learning phase retained fewer inferred meanings than those that started with the Executive Functioning (EF) task and even those that directly started with the learning phase. ToM and EF are often correlated in the context of pragmatic processing (Apperly, 2012; Carlson & Moses, 2001; Fairchild & Papafragou, 2021; Hughes & Ensor, 2007; Kampa et al., 2025; Nilsen & Graham, 2009), as representations are held and manipulated in working memory, or as selection between literal and pragmatically enriched interpretations is performed by cognitive control. But our findings provide a clear dissociation and suggest that pragmatic inference in the context of our study relies more on social cognition, rather than cognitive control. It is likely that the assumption that the speaker seeks to be informative does not require listeners to actively resolve conflicts between their own belief and the speaker’s belief and thus may not engage cognitive control in the form of inhibition to the same extent as in other pragmatic work. Furthermore, as EF covers a multitude of constructs, others may play additional roles in modulating the relationship between ToM, pragmatic processing, and word learning. As such, future work should seek to tease apart the impact of other EF domains. Note that further study with additional ToM tasks is similarly warranted to fully validate and replicate this relationship. While participants’ performance in the Mind-in-the-Eyes task shows promising relationship with word learning, this task at best only reflects one of many dimensions of mentalization, i.e., mentalizing about affective states (Quesque et al., 2024).

Our work found a modulating effect of ToM usage. However, our current paradigm is unable to fully differentiate between a taxing effect on pragmatic inference retention, placing it more in line with work such as Smalle et al. (2022), which sought to deplete the target resource, or a boosting effect in direct mapping retention, in line with studies such as Hsu and Novick (2016), who sought to boost it via pre-emptive engagement. However, regardless of the interpretation, this speaks to ToM not just as a construct manipulable on a state level, but one that has limits, either from individual differences in traits or in-the-moment state, on its cognitive capacity that stretches beyond its immediate engagement and into how it interfaces with other cognitive systems. Interestingly, the degree of modulating effect was not proportional to the ToM task demands: engaging in a shorter ToM task prior to word learning did not result in a perturbation of meaning retention. These results provide an interesting complement to studies exploring cognitive control and its relation to sentence processing (e.g., Hsu & Novick, 2016). In these studies, participants’ syntactic parsing was facilitated when they were briefly engaged in an inhibitory control task (e.g., Stroop) prior to syntactic disambiguation. Together with our results, such cognitive priming effects are particularly valuable for elucidating the underlying cognitive constituents for language processing or learning. However, a critical distinction between work such as Hsu and Novick (2016) and our paradigm must be noted—unlike in such cognitive control studies, where priming effects are interweaved and take place on a time scale of milliseconds/seconds, our design is blocked, with both initial priming and priming effects extending on the timescale of minutes. This places it more in line with prior social-engagement-focused work (e.g., Hopkins & Branigan, 2020). As such, the effects of social-cognitive engagement are shown to be lasting on the scale of mental state rather than fleeting. How this is similar to or differs from other cognitive constituents that support learning as well as how this plays out on a neuronal level necessitates further exploration, as does work focusing on elucidating the temporal scale of ToM engagement.

What exactly is the role of ToM in retention? One possibility is that items encoded via ToM have a privileged status in memory. Lin et al. (2019) found that socially important information, such as faces, was more precisely retrieved compared to non-social information, such as scenes. Similarly, the memory advantage reported in the current study might also suggest that information learned through heavily engaging the ToM system carries greater weight to ensure communication success, compared to information gained through less socially taxing learning. Another possibility is that better ToM skills require fewer resources devoted to the initial computation and thus free up additional resources that can be allotted to active memory encoding. This could explain the possible taxing effect seen within our Experiment 2, as manipulating ToM capacity in our preceding task did not seem to affect initial mapping, but only affected subsequent recall/retention. However, neither of these possibilities touch on one crucial question: what aspect of ToM is most critical to our outcome? Pragmatic inference resolution in our experiment can be divided into two steps: first, the process of computing a speaker’s intention and second, the active process of using this computation to select the intended referent and reject a competitor. To determine the role that each stage may be playing in facilitating retention, further experimental work would need to tease apart these two aspects from one another. This could be accomplished by either investigating retention via a type of disambiguation that does not involve social cognition, by manipulating the demands of social cognition in the inference condition, or by studying individuals with impaired social communication skills.

Be that as it may, our data bear on the current debate on the role of ToM in pragmatic inference, and more specifically, those inferences that are driven by informativeness calculations. Unlike suggestions that the role of ToM in such contexts may be limited (Andrés-Roqueta & Katsos, 2020; Ferguson et al., 2018; Hochstein et al. 2018; Kissine, 2012; Noveck et al., 2007; Pijnacker et al., 2009; Wampers et al., 2018), our data support the theoretical position that pragmatic processes during communication rely on mentalizing (Clark, 1990; Grice, 1975; Sperber & Wilson, 1986). Furthermore, our results concur with other findings that point to contributions of ToM on pragmatic calculations in adults (Bambini et al., 2011; Bašnáková et al., 2014; Fairchild & Papafragou, 2021; Hornick & Shetreet, 2022; Kampa et al., 2025; Spotorno et al., 2012), and suggest that consideration of other people’s intentions or knowledge can facilitate word learning in children (Happé & Loth, 2002; Houston-Price et al., 2010; Jaswal, 2004; Saylor & Carroll, 2009, among many others). A study closely related to the present paradigm but with a different ToM measure, Trice et al. (2025), further supports the role of ToM in children’s word learning and retention. However, further work is necessary to determine whether the underlying mechanisms are consistent across development and into adulthood or shift as the ToM system fully matures.

### Constraints on Generality and Further Directions

First, when considering individual difference measures, future work should focus on relations to non-linguistics ToM tasks to provide evidence for a relationship more clearly independent of language abilities. Furthermore, our chosen EF task is specific to inhibitory control. This was a deliberate choice. As our pragmatic inference condition uniquely has the need to need to compare between distinct competitors, the lack of relationship between inhibitory control and retention outcomes for pragmatic inference lessens the likelihood that it is the presence and suppression of this competitor that results in better memory outcomes. However, many other categories of executive functioning such as attention and working memory likely also contribute to our results (Ackermann et al., 2017). Thus, we cannot conclude that EF does not modulate the retention advantage seen, and future work should take care to systematically examine the role of different types of executive functions.

Our experimental design also features linguistic asymmetries. We used identical syntactic structures for the first sentence of each condition (e.g., “Look! I like this ___”) to control for effects of desire for a specific initially-ambiguous object on social and task engagement. However, this meant that the novel word in the direct mapping condition preceded by a definite determiner *this*, but in the pragmatic inference condition was proceeded by an indefinite determiner *a* (e.g., “…It’s holding a ___”). This results in the novel object for the direct mapping condition being the target. This may lead to increased saliency and attention over the pragmatic inference condition, where the novel object disambiguates between the possible targets, but it not itself what the participants are asked to select. However, if this saliency had an effect, we would’ve expected better memory for directly mapped words, suggestion that the effects of pragmatic inference at least had more weight. Future work should further explore the effects of specific determiner usage on word learning.

In reference to Experiment 2 in particular, it is important to note that, as our comparison was between-group for the effect of ToM modulation rather than within-group, we do not have a within-group baseline metric for memory of novel words to control between groups. This complicates our ability to interpret findings based on within-context accuracy differences between groups. Incidental differences in memory capacity, as well as task engagement, language and cognitive skills, may all modulate the raw accuracy for a given group and create a confound for between-group accuracy comparisons. Instead, our strongest conclusions are predicated on the relative *degree* of within-subject retention success for pragmatic inference vs direct mapping. Thus, we cannot say for certain that the lack of significant difference between directly mapped and pragmatically inferred words after ToM priming comes from a decrease in the number of pragmatically inferred words that would otherwise have been remembered by these specific individuals as opposed to an increase in directly mapped words or a mix of the two. It is also possible that downstream effects of the ToM engagement boosted memory for directly mapped words but interfered when the over-taxed resources were needed for pragmatic inference mapping or that a ceiling effect was seen. Within-subject work will be needed to definitively tease apart such possibilities and provide stronger support for potential causality.

Finally, it should be noted throughout both experiments the target population was young adults, specifically U.S.A.-based graduate students, who were monolingual native English speakers. This population was chosen to control for possible age and maturation effects of our variables of interests along with any impacts of bilingualism as well as for the sake of experimenter convenience. Future replications should ensure these results hold in the more general population as well as with monolingual speakers of other languages, and expansions of this work should determine if age, development, bi- or multi-lingual status, or clinical subgroup impact the generalizability of these findings.

## Acknowledgment

A special thanks to our participants for their participation in our study. Hannah Fox and Krystal Mendez were the voices for the stimuli.

## Funding Information

This research was supported by internal funds from the University of Delaware and Northeastern University awarded to Zhenghan Qi.

## Author Contributions

K.T.: Conceptualization; Formal analysis; Investigation; Visualization; Writing – original draft; Writing – review & editing. D.S.: Conceptualization; Formal analysis; Investigation; Methodology; Visualization; Writing – original draft; Writing – review & editing. A.P.: Conceptualization; Methodology; Supervision; Writing – review & editing. Z.Q.: Conceptualization; Investigation; Methodology; Resources; Supervision; Visualization; Writing – review & editing.

## Data Availability Statement

All experimental stimuli, data, and analytical scripts used in the current study are publicly available in the OSF repository: https://osf.io/qy7bg/overview?view_only=830246afb8944a248b0a1155cb2be269.

## Note

^1^ Critical changes were: 1) Removing pointing cues to a) situate listeners in a referential disambiguation task with word learning as a byproduct to elicit complex pragmatic reasoning and represent learning situations frequently present in adulthood real-world environments and b) decouple gestural processing/integration from linguistic pragmatic reasoning, ToM, and inferencing of speaker intent; 2) Using completely novel objects, as existing labels for objects (e.g., hat or bandana) might interfere with the learning process for new labels (Coutanche & Thompson-Schill, 2014); 3) Comparing effects of inference vs. direct mapping on word learning rather than focusing on inference.

## Supplementary Material



## Appendix

**Table A1.** Individual difference measurements and their standard errors for all experimental groups.

**Group**	**ToM**	***SE***	**Flanker RT (ms) (Incongruent – Congruent)**	***SE***	**Wisconsin Card Sorting**	***SE***
Learning-First-Expt 1	0.62	0.02	22	45	–	–
Learning-First-Expt 2	0.70	0.02	32	5	–	–
EF-First	0.63	0.02	53	38	0.48	0.02
ToM-full-First	0.66	0.02	28	16	0.47	0.02
ToM-partial-First	0.62	0.02	10	10	0.42	0.02

**Table A2.** Descriptive stastistics for accuracy across all experiments and tasks.

**Conditions**	**Mean**	***SE***	**Split-Half**
Experiment 1			
Immediate Recall: Direct Mapping	0.55	0.08	0.72
Immediate Recall: Inference	0.65	0.08	0.67
Retention: Direct Mapping	0.51	0.08	0.76
Retention: Inference	0.65	0.08	0.72
Experiment 2 – Learning First			
Immediate Recall: Direct Mapping	0.59	0.08	0.70
Immediate Recall: Inference	0.75	0.07	0.63
Retention: Direct Mapping	0.53	0.08	0.72
Retention: Inference	0.61	0.08	0.71
Experiment 2 – EF First			
Immediate Recall: Direct Mapping	0.55	0.08	0.59
Immediate Recall: Inference	0.61	0.08	0.59
Retention: Direct Mapping	0.50	0.08	0.49
Retention: Inference	0.60	0.08	0.45
Experiment 2 – ToM-full-First			
Immediate Recall: Direct Mapping	0.65	0.08	0.67
Immediate Recall: Inference	0.62	0.08	0.66
Retention: Direct Mapping	0.60	0.08	0.70
Retention: Inference	0.55	0.08	0.73
Experiment 2 – ToM-partial-First			
Immediate Recall: Direct Mapping	0.48	0.08	0.58
Immediate Recall: Inference	0.61	0.08	0.51
Retention: Direct Mapping	0.51	0.08	0.73
Retention: Inference	0.59	0.08	0.67

**Table A3.** Alpha and split-half reliability for mind-in-the-eyes across groups.

**Group**	**Split-Half**	**95% CI**	***α***	**95% CI**
Learning-First-Expt 1	0.57	0.34−75	0.61	0.40−0.77
Learning-First-Expt 2	0.52	0.28−0.72	0.61	0.40−0.77
EF-First	0.60	0.39−0.77	0.66	0.48−0.80
ToM-full-First	0.47	0.19−0.69	0.54	0.30−0.73
ToM-partial-First	0.53	0.28−0.73	0.61	0.40−0.77

*Note*. ToM-partial-First has only 14 trials rather than the original 36, and as such differences in alpha and split-half reliability should not be treated as comparable to the others or representative of the task more generally. CI = Confidence Interval; *α* = Cronbach’s *α*. The CI for uses the Feldt et al. (1987) procedure.

**Table A4.** Parameter estimates for recall and retention accuracy scores for Experiment 1.

**Effects**	**Fixed**			**Random**	
	**Estimate**	***SE***	***Z***	**Variance**	***SD***
*Immediate Recall Task*					
Intercept	−0.43	0.37	−1.16		
Context (Direct Mapping vs Inference)	0.84	0.41	2.05*		
Learning Score	1.17	0.35	3.33***		
ID				0.90	0.95
Context				1.67	1.29
Item				0.28	0.52
*Retention Task*					
Intercept	−0.43	0.33	−1.30		
Context (Direct Mapping vs Inference)	1.02	0.35	2.90**		
Learning Score	1.08	0.33	3.30***		
ID				0.94	0.97
Context				2.50	1.59
Item				0.00	0.00

*Note*. ID and Item are random intercepts and context a random slope. Significance levels: . *p* < 0.1, **p* < .05, ***p* < .01, ****p* < .001.

**Table A5.** Parameter estimates for the multiple linear regression models predicting word recall and retention across learning contexts (Experiment 1).

**Immediate Recall**	**Estimate**	***SE***	***t***
*Inference*			
Intercept	0.41	0.24	1.74.
Theory of Mind	0.01	0.01	1.12
Executive Function	0.00	0.00	−1.79

*Direct Mapping*			
Intercept	0.11	0.25	0.45
Theory of Mind	0.02	0.01	1.83
Executive Function	−0.00	0.00	−1.43

**Retention**	**Estimate**	***SE***	***t***
*Inference*			
Intercept	−0.16	0.17	−0.90
Immediate Recall	0.66	0.12	5.36***
Theory of Mind	0.02	0.01	2.32*
Executive Function	−0.00	0.00	−1.10

*Direct Mapping*			
Intercept	−0.23	0.19	−1.16
Immediate Recall	0.71	0.13	5.36***
Theory of Mind	0.02	0.01	1.72
Executive Function	0.00	0.00	0.61

*Note*. Significance levels: . *p* < .1, **p* < .05, ***p* < .01, ****p* < .001.

**Table A6.** Parameter estimates for learning in the inferred condition across tasks between groups (Experiment 2).

**Effects**	**Fixed**			**Random**	
	**Estimate**	***SE***	***Z***	**Variance**	***SD***
*Learning Task –ToM-full Baseline*					
Intercept	1.09	0.52	2.08*		
Contrast 1 (ToM-full-First vs Learning-First)	0.11	0.46	0.25		
Contrast 2 (ToM-full-First vs EF-First)	0.83	0.46	1.79		
Contrast 3 (ToM-full-First vs ToM-partial-First)	0.11	0.46	0.24		
ID				3.13	1.77
Item				2.86	1.70
					
*Learning Task –Learning-First Baseline*					
Intercept	1.20	0.52	2.30*		
Contrast 1 (Learning-First vs ToM-full-First)	−0.11	0.46	−0.24		
Contrast 2 (Learning-First vs EF-First)	0.71	0.46	1.54		
Contrast 3 (Learning-First vs ToM-partial-First)	−0.00	0.46	−0.00		
ID				3.13	1.77
Item				2.86	1.69
					
*Learning Task –ToM-full Baseline plus ToM Task*				
Intercept	1.72	1.13	1.53		
Contrast 1 (ToM-full-First vs Learning-First)	0.17	0.47	0.37		
Contrast 2 (ToM-full-First vs EF-First)	0.80	0.46	1.72		
Contrast 3 (ToM-full-First vs ToM-partial-First)	0.09	0.46	0.20		
ToM Accuracy	−0.99	1.56	−0.63		
ID				3.12	1.77
Item				2.86	1.69
					
*Learning Task –Learning-First Baseline plus ToM Task*				
Intercept	1.90	1.22	1.56		
Contrast 1 (Learning-First vs ToM-full-First)	−0.18	0.47	−0.37		
Contrast 2 (Learning-First vs EF-First)	0.62	0.58	1.28		
Contrast 3 (Learning-First vs ToM-partial-First)	−0.08	0.47	−0.18		
ToM Accuracy	−0.99	1.56	−0.63		
ID				3.12	1.77
Item				2.86	1.69

*Note*. ID and Item are random intercepts, Significance levels: . *p* < .1, * *p* < .05, ** *p* < .01, *** *p* < .001.

**Table A7.** Parameter estimates for immediate recall accuracy between groups (Experiment 2).

**Effects**	**Fixed**			**Random**	
	**Estimate**	***SE***	***Z***	**Variance**	***SD***
*Immediate Recall Task –ToM-full Baseline*					
Intercept	0.40	0.19	2.12*		
Context (Direct mapping vs. Inference)	0.31	0.33	0.94		
Contrast 1 (ToM-full-First vs Learning-First)	0.53	0.24	2.21*		
Contrast 2 (ToM-full-First vs EF-First)	0.29	0.24	1.21		
Contrast 3 (ToM-full-First vs ToM-partial-First)	−0.16	0.24	−0.67		
Context * Contrast 1	0.59	0.39	1.50		
Context * Contrast 2	−0.48	0.39	−1.24		
Context * Contrast 3	0.32	0.38	0.84		
ID				0.69	0.83
Context				1.38	1.18
Item				0.13	0.36
*Immediate Recall Task - ToM-full Baseline plus ToM Task*				
Intercept	−0.77	0.52	−1.48		
Context (Direct mapping vs. Inference)	0.30	0.33	0.93		
Contrast 1 (ToM-full-First vs Learning-First)	0.35	0.25	1.41		
Contrast 2 (ToM-full-First vs EF-First)	0.24	0.23	1.04		
Contrast 3 (ToM-full-First vs ToM-partial-First)	−0.18	0.23	−0.76		
Context * Contrast 1	0.59	0.39	1.49		
Context * Contrast 2	−0.48	0.39	−1.25		
Context * Contrast 3	0.32	0.38	0.83		
ToM Accuracy	1.92	0.80	2.41*		
ID				0.65	0.80
Context				1.39	1.18
Item				0.13	0.36

*Note*. ID and Item are random intercepts and context a random slope. Significance levels: . *p* < .1, *.05, ** *p* < .01, *** *p* < .001.

**Table A8.** Parameter estimates for retention accuracy (Experiment 2).

**Effects**	**Fixed**			**Random**	
	**Estimate**	***SE***	***Z***	**Variance**	***SD***
*Retention Task –ToM-full Baseline*					
Intercept	0.41	0.21	1.94		
Context (Direct mapping vs. Inference)	−0.29	0.30	−0.95		
Contrast 1 (ToM-full-First vs Learning-First)	−0.04	0.27	−0.15		
Contrast 2 (ToM-full-First vs EF-First)	−0.15	0.27	−0.56		
Contrast 3 (ToM-full-First vs ToM-partial-First)	−0.12	0.27	−0.45		
Context * Contrast 1	0.74	0.35	2.13*		
Context * Contrast 2	0.77	0.34	2.23*		
Context * Contrast 3	0.72	0.35	2.06*		
ID				1.02	1.01
Context				0.84	0.92
Item				0.12	0.34
*Retention Task – Learning-First Baseline*					
Intercept	0.37	0.21	1.74		
Context (Direct mapping vs. Inference)	0.46	0.30	1.52		
Contrast 1 (Learning-First vs ToM-full-First)	0.04	0.27	0.15		
Contrast 2 (Learning-First vs EF-First)	−0.11	0.27	−0.41		
Contrast 3 (Learning-First vs ToM-partial-First)	−0.08	0.27	−0.29		
Context * Contrast 1	−0.74	0.35	−2.23*		
Context * Contrast 2	0.02	0.34	0.07		
Context * Contrast 3	−0.03	0.34	−0.08		
ID				1.02	1.01
Context				0.84	0.92
Item				0.12	0.34
					
*Retention Task – ToM-full Baseline plus ToM Task*				
Intercept	−1.13	0.62	−1.84		
Context (Direct mapping vs. Inference)	−0.29	0.30	−0.96		
Contrast 1 (ToM-full-First vs Learning-First)	−0.21	0.28	−0.75		
Contrast 2 (ToM-full-First vs EF-First)	−0.09	0.27	−0.34		
Contrast 3 (ToM-full-First vs ToM-partial-First)	−0.08	0.27	−031		
Context * Contrast 1	0.75	0.35	2.14*		
Context * Contrast 2	0.77	0.35	2.24*		
Context * Contrast 3	0.71	0.35	2.05*		
ToM Accuracy	2.42	0.91	2.65**		
ID				0.95	0.97
Context				0.84	0.92
Item				0.12	0.34
*Retention Task – Learning-First Baseline plus ToM Task*				
Intercept	−1.33	0.67	−1.98*		
Context (Direct Mapping vs. Inference)	0.46	0.30	1.52		
Contrast 1 (Learning-First vs ToM-full-First)	0.21	0.28	0.75		
Contrast 2 (Learning-First vs EF-First)	0.11	0.28	0.41		
Contrast 3 (Learning-First vs ToM-partial-First)	0.12	0.28	0.44		
Context * Contrast 1	−0.75	0.35	−2.13*		
Context * Contrast 2	0.03	0.34	0.08		
Context * Contrast 3	−0.03	0.35	−0.09		
ToM Accuracy	2.42	0.91	2.65**		
ID				0.95	0.97
Context				0.84	0.92
Item				0.12	0.34

*Note*. ID and Item are random intercepts and context a random slope. Significance levels: . *p* < .1, * *p* < .05, ** *p* < .01, *** *p* < .001.
