# Supplementary material for: Pragmatics and Social Cognition in Learning and Remembering Words
Source: Open Mind (Camb). 2026 Sep 5;10:1432–69. doi: 10.1162/OPMI.a.383 (PMC13682635; doi:10.1162/OPMI.a.383)
Supplement: Supplementary file 1 [file opmi-10-1432-s001.pdf]

Supplementary Materials

1. Analysis Details

1.1. Power Analysis

A sample size of 36 participants was determined based on some preliminary data from a pilot study not included in these experiments. Here, we used the correlation coefficient for the relationship between participants' Theory of Mind score and their performance in successfully recalling inferred meaning – 0.52 – to calculate the desired sample size to detect a significant relationship (95% power, α=0.05) between the two variables as our main analysis of interest (GPower 3.1).

1.2. Experiment 1

1.2.1 *Pragmatic vs. direct-mapping contexts in word learning and retention*

To examine performance during learning, immediate recall, and retention, trial-by-trial binomial generalized linear mixed effects models were constructed (*glmer*) were constructed for each of these three timepoints using the lme4 package of R (Baayen 2008; Baayen et al. 2008).

For learning, as learning performance between the two contexts would not be directly compared, separate linear models were constructed for each context. The binomial trial-by-trial mapping accuracy for the learning scores was the dependent variable. As the purpose was to determine whether performance was above-chance, no fixed effects were included. By-item and by-participant random intercepts were used.

Subsequently, participants' responses were coded as correct (1) or incorrect (0) depending on whether they selected the correct object for each novel label they heard. For each item, we calculated a learning score marking the item as learned (coded as 1) only if participants chose the correct referent in both learning trials. Otherwise, the item was marked as not learned, coded as 0. These learning scores were entered as a binomial predictor in the models assessing the context effect for immediate recall and retention, described below.

For immediate recall and retention, context type (inferred vs direct mapping) was the fixed effect and learning score as the covariate. Trial-by-trial binomial accuracy data during the given timepoint (immediate recall or retention) was the dependent variable. Random intercepts were included for items and participants. Additionally, random slopes for context type were included. Since the fixed predictor of context type had two levels, its effect was assessed with one planned comparison between the direct mapping contexts and inferred (contrast coding: 0.50, -0.50) in order to use the mean across the different levels for the given effect as the baseline.

1.2.2 *Relationship between Theory of Mind (ToM) and word memory*

Multiple linear regression models were created for immediate recall and retention and for each learning context (direct mapping or inference). The proportion of correct responses was entered

as the dependent variable with ToM scores and EF scores entered as predictors. For the models predicting word retention, we additionally controlled for participants' performance on the immediate recall task for the corresponding context.

1.3. Experiment 2

For within-group models, the construction of each model followed the same procedures as laid out in 1.2.1, with the dependent variable being restricted to the trial-bi-trial binomial accuracy for the given group.

For between-group models, trial-by-trial binomial generalized linear mixed effects models (glmer) were built for learning, immediate recall, and retention.

For learning, contexts were again examined separately, with the binomial trial-by-trial mapping accuracy for the learning scores was the dependent variable. Group served as the fixed effect. Here, treatment coding was used, with our primary group of interest as the baseline (0) being compared to each of the remaining groups (1). Thus, for the initial model, ToM-full-First was coded as 0 and compared to Learning-First (1), EF-First (1), and ToM-partial-First (1). If group differences were seen between ToM-full-First and either EF-First or ToM-partial-First, an additional model with Learning-First coded as (0) and compared to each other group was created. To ensure that any confounding effects of ToM difference between groups were accounted for, models will be replicated with Mind-in-the-Eyes score as a covariate. Random intercepts were included for items and participants.

For immediate recall and retention, similar setups to learning were used – binomial trial-by-trial mapping accuracy being predicted for the given timepoint based on group using treatment coding, with subsequent models altering the baseline if necessitated and including Mind-in-the-Eyes score as a covariate. Here, however, context type (inferred vs direct mapping) served as an additional fixed effect and its interaction with group was examined. Contrast coding (0.50, -0.50) was used for context, just as in within-group models. In addition to the random intercepts for items and participants, random slopes for context type were included.

2. Results

2.1 Individual Difference Measures vs Immediate Recall and Retention Accuracy

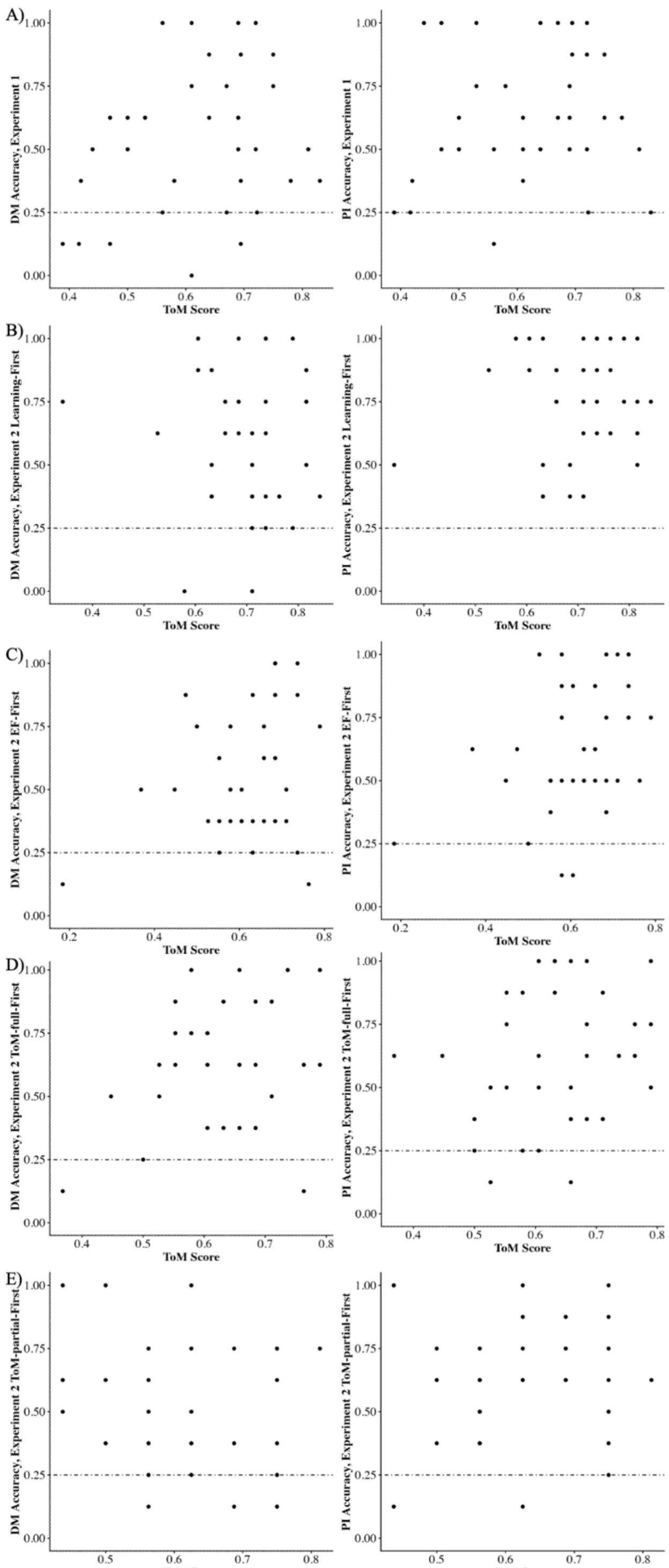

Figure 1: Scatterplots of ToM and Mean Immediate Recall Accuracy for A) Experiment 1, B) Experiment 2 Learning First, C) Experiment 2 EF-First, D) Experiment 2 ToM-full-First and 3) ToM-partial-First. First column correlates ToM with Direct Mapping (DM) accuracies, second with Pragmatic Inference (PI) accuracies. ToM=Theory of Mind, as measured by accuracy in the Mind-in-the-Eyes Task.

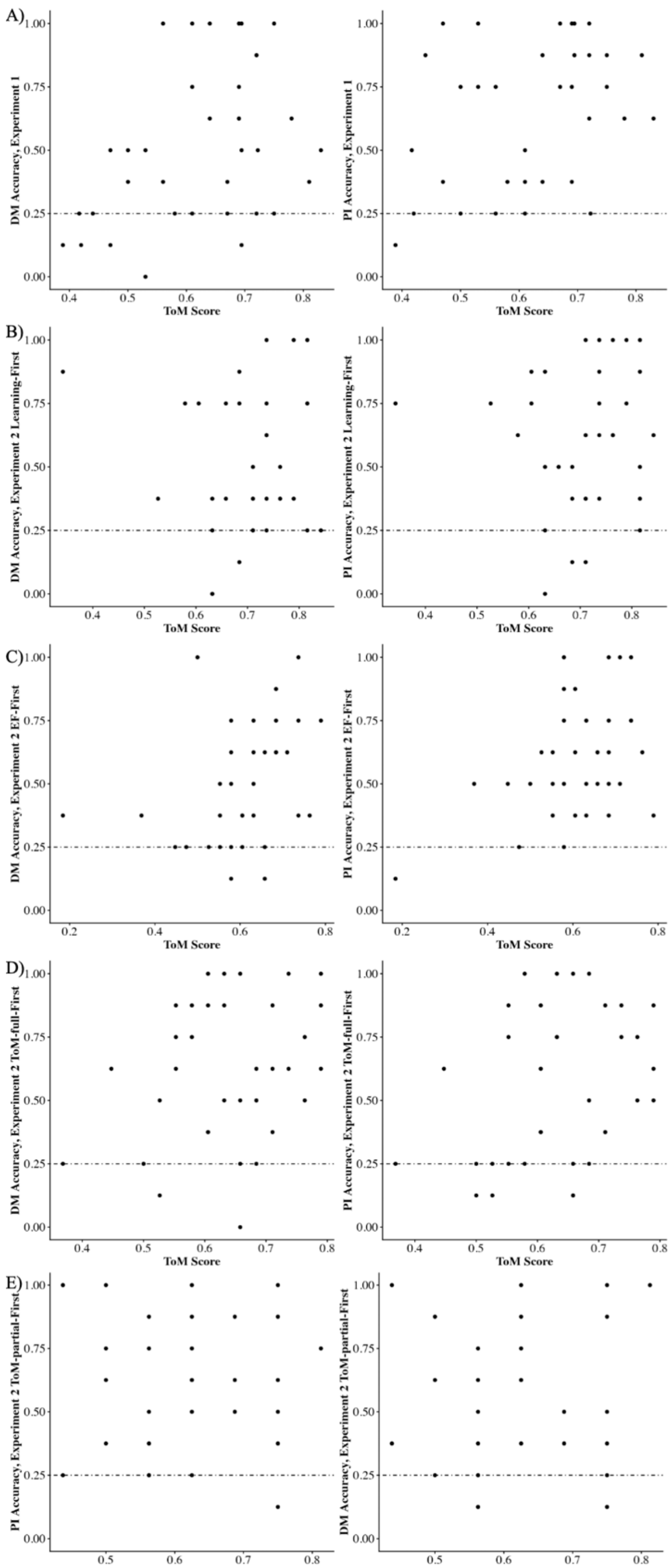

Figure 2: Scatterplots of ToM and Mean Retention Accuracy for A) Experiment 1, B) Experiment 2 Learning First, C) Experiment 2 EF-First, D) Experiment 2 ToM-full-First and 3) ToM-partial-First. First column correlates ToM with Direct Mapping (DM) accuracies, second with Pragmatic Inference (PI) accuracies. ToM=Theory of Mind, as measured by accuracy in the Mind-in-the-Eyes Task.

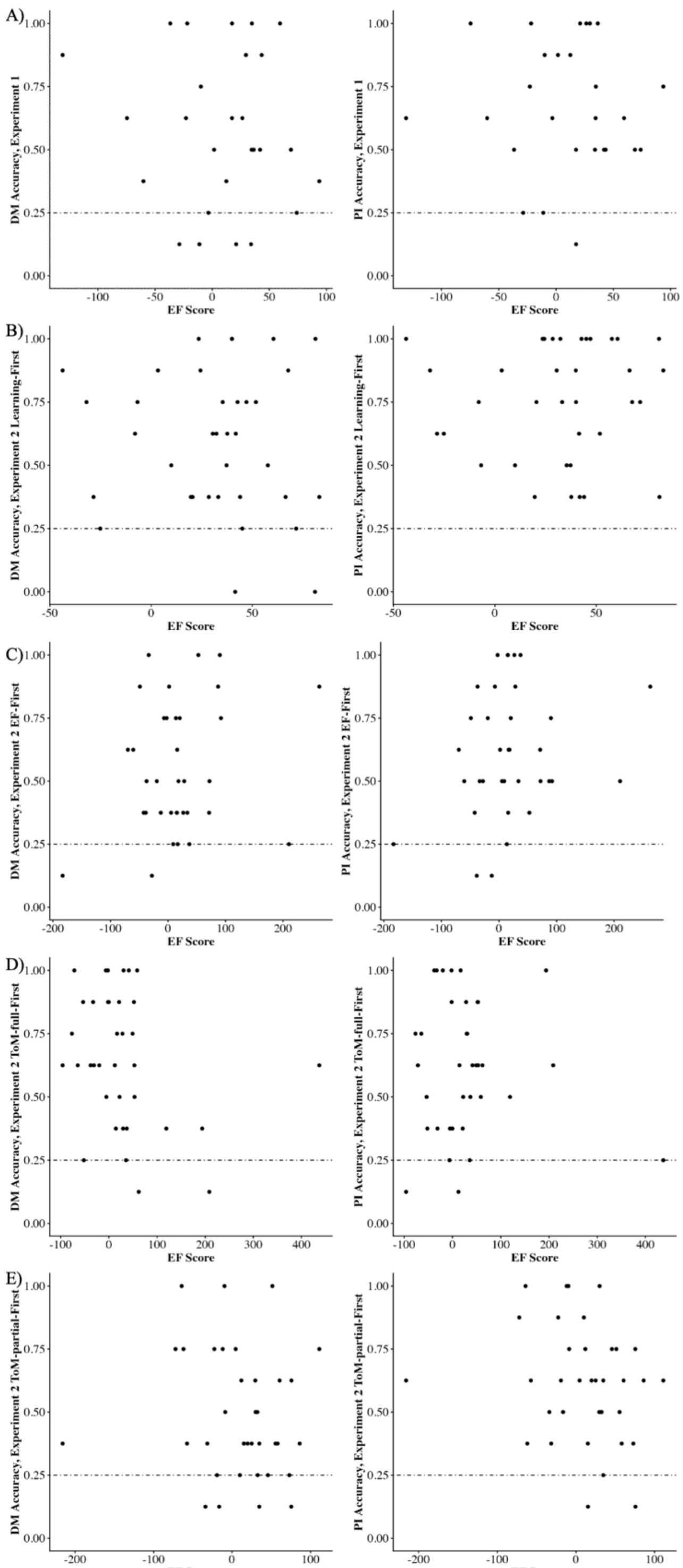

Figure 3: Scatterplots of EF and Mean Immediate Recall Accuracy for A) Experiment 1, B) Experiment 2 Learning First, C) Experiment 2 EF-First, D) Experiment 2 ToM-full-First and 3) ToM-partial-First. First column correlates EF with Direct Mapping (DM) accuracies, second with Pragmatic Inference (PI) accuracies. EF=Executive Functioning, as measured by incongruent – congruent reaction time in a Flanker Task.

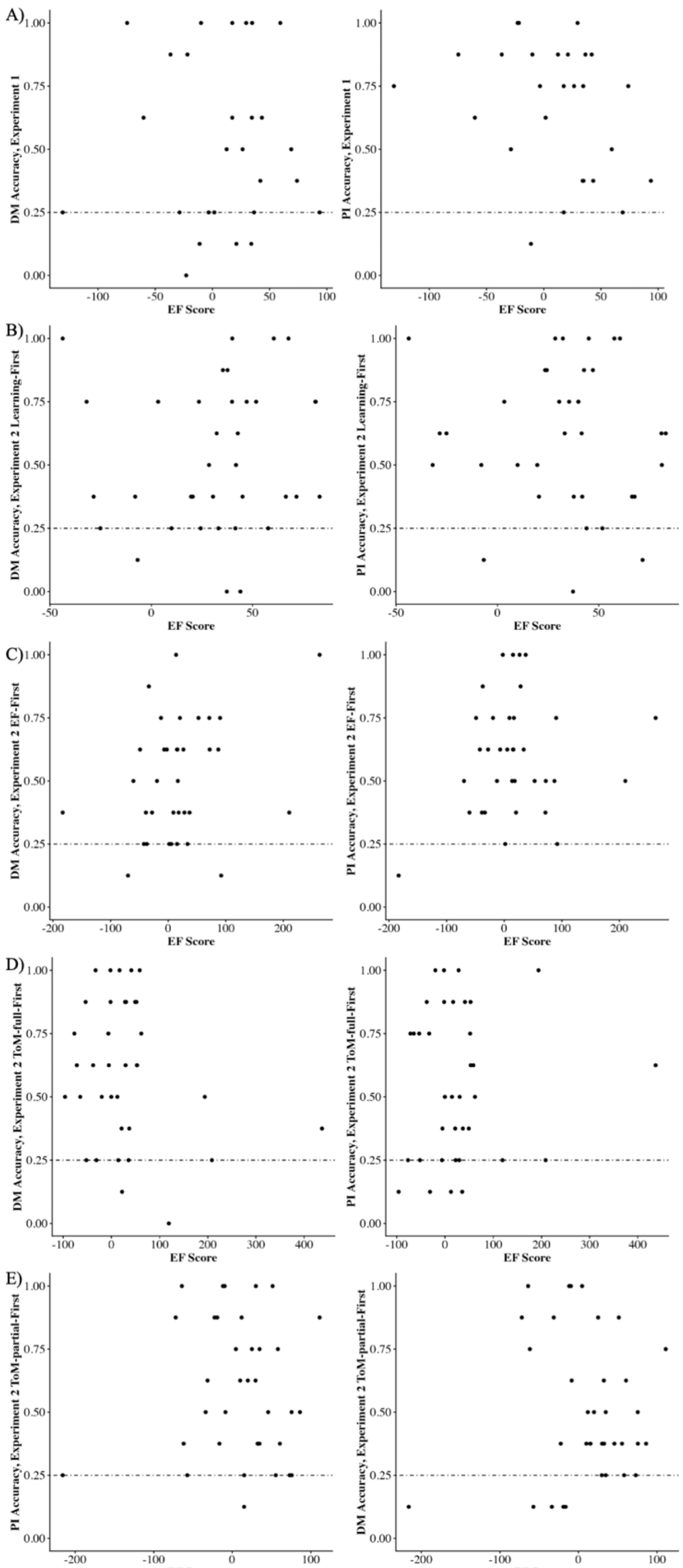

Figure 4: Scatterplots of EF and Mean Retention Accuracy for A) Experiment 1, B) Experiment 2 Learning First, C) Experiment 2 EF-First, D) Experiment 2 ToM-full-First and 3) ToM-partial-First. First column correlates EF with Direct Mapping (DM) accuracies, second with Pragmatic Inference (PI) accuracies. EF=Executive Functioning, as measured by incongruent – congruent reaction time in a Flanker Task.
